# A robust and light-weight transfer learning-based architecture for accurate detection of leaf diseases across multiple plants using less amount of images

**DOI:** 10.3389/fpls.2023.1321877

**Published:** 2024-01-11

**Authors:** Md. Khairul Alam Mazumder, M. F. Mridha, Sultan Alfarhood, Mejdl Safran, Md. Abdullah-Al-Jubair, Dunren Che

**Affiliations:** ^1^Department of Computer Science, American International University-Bangladesh, Dhaka, Bangladesh; ^2^Department of Computer Science, College of Computer and Information Sciences, King Saud University, Riyadh, Saudi Arabia; ^3^School of Computing, Southern Illinois University, Carbondale, IL, United States

**Keywords:** multi-leaf disease, plant leaf disease, multi-plant leaf disease, attentive-transition, attention module, lightweight architecture, robust architecture, swish activation

## Abstract

Leaf diseases are a global threat to crop production and food preservation. Detecting these diseases is crucial for effective management. We introduce LeafDoc-Net, a robust, lightweight transfer-learning architecture for accurately detecting leaf diseases across multiple plant species, even with limited image data. Our approach concatenates two pre-trained image classification deep learning-based models, DenseNet121 and MobileNetV2. We enhance DenseNet121 with an attention-based transition mechanism and global average pooling layers, while MobileNetV2 benefits from adding an attention module and global average pooling layers. We deepen the architecture with extra-dense layers featuring swish activation and batch normalization layers, resulting in a more robust and accurate model for diagnosing leaf-related plant diseases. LeafDoc-Net is evaluated on two distinct datasets, focused on cassava and wheat leaf diseases, demonstrating superior performance compared to existing models in accuracy, precision, recall, and AUC metrics. To gain deeper insights into the model’s performance, we utilize Grad-CAM++.

## 1 Introduction

The increasing global population and the subsequent rise in the demand for agricultural products have posed growing challenges to the role of agriculture in advancing sustainable development (Wang et al., 2022) and guaranteeing global food security. As a substantial driver of the global economy, agriculture is a fundamental provider of food, income, and employment opportunities (Eunice et al., 2022). Given the limited scope for expanding cultivable land, the sole means of augmenting agricultural production is enhancing land productivity (Gebbers and Adamchuk, 2010). Cassava and wheat are essential crops that play significant roles in global agriculture and food security. Cassava, a botanical species, has leaves that exhibit a notable quantity of protein and vitamins (Sambasivam and Opiyo, 2021). Cassava stands as a prominent staple crop in Africa. Cultivation of cassava is prevalent in three continents, namely Africa, Asia, and Latin America, which collectively serve as key contributors to the global production of cassava. Cassava has a comparative advantage over yam and other African grains, roots, and tubers because it can flourish in various soil conditions and adapt to multiple climatic circumstances. This adaptability facilitates the successful production of cassava across numerous geographical regions. Along with the nutritional content of rice, it provides numerous health advantages. However, cassava production has experienced a decline as a result of various diseases that affect the crop, including mosaic disease, brown streak disease, and bacterial blight (Zhong et al., 2022). Moving on to wheat is a prominent global crop and a significant staple in the human diet. It is cultivated in the largest planting area globally, and the yield of this crop plays a crucial role in ensuring food security for most countries worldwide. Wheat has considerable nutritional value, encompassing a wide range of carbohydrates, fats, proteins, and other vital substances necessary for human sustenance. Diseases have a significant impact on both the yield and quality of wheat. The decrease in wheat productivity results in financial implications and threatens human well-being. Wheat powdery mildew, wheat leaf blight, and wheat rust are commonly observed and highly detrimental diseases affecting wheat crops (Kloppe et al., 2022).

According to projections, the world population is anticipated to surpass 9 billion by the year 2050. Consequently, there will be a corresponding doubling of food consumption, necessitating a minimum increase of 70% in production to meet the heightened demand (Gebbers and Adamchuk, 2010). The issue of food security (Wang et al., 2022), both at the local and global level (Eunice et al., 2022), is a significant and fundamental challenge that must be addressed to achieve sustainable human development (Mustak Un Nobi et al., 2023). Therefore, it is imperative to prioritize a sustainable environment, the absence of diseases, and the attainment of high quality and high yield (Wang et al., 2022) as essential for enhancing food production to meet future demands (Eunice et al., 2022). Several contemporary approaches rely on computer vision methods in plant science, facilitating the surveillance of various agricultural sectors, including banana, wheat, black gram, tomato, maize, grape, citrus, potato, rice, cassava, etc. Researchers worldwide actively employ machine learning and computer vision techniques to detect plant leaf diseases effectively (Gebbers and Adamchuk, 2010). In agriculture, monitoring the health of plants is very important to achieve the desired crop yield. Plant disease significantly threatens food security and the annual revenue growth of the agricultural industry (Eunice et al., 2022). The task of classifying plant leaf diseases presents significant challenges due to the presence of high inter-class similarities and complicated pattern variations. The application of deep learning approaches (Mustak Un Nobi et al., 2023) to identify plant diseases has emerged as a pivotal component in the observation and evaluation of the production of distinct plant species. The rapid advancement in high-performance computing and image processing components has enabled the effective utilization of deep learning techniques in diverse domains (Fan and Guan, 2023). These methods (Bajpai et al., 2023) have proven highly proficient in uncovering complex structures within high-dimensional data, making them applicable to a wide array of fields, including science, engineering, industry, bioinformatics, and agriculture (Pal and Kumar, 2023). Deep learning methods demonstrate superior performance compared to traditional classification networks in the real-time detection of plant leaf diseases (Shah et al., 2022). The conventional machine learning approach (Wang and Su, 2022) exhibits a limited generalization capacity and requires the manual extraction of disease-related features. The emergence of deep learning provides an additional approach to establishing disease detection (Eunice et al., 2022). The Convolutional Neural Network (CNN) is widely recognized as one of the most prominent techniques in the field of deep learning (Wu et al., 2022a). CNN can extract distinctive characteristics from images that exhibit diverse scales autonomously (Eunice et al., 2022). CNN extracts intricate, low-level features from images (Wang and Su, 2022). Therefore, CNN is favored over conventional approaches in the automated identification of plant diseases due to their superior performance(Eunice et al., 2022). CNN demonstrates significant capabilities in effectively segmenting image components with high accuracy. In recent years, agriculture has utilized it extensively (Li et al., 2021b). This research presents a state-of-the-art deep learning architecture based on transfer learning for detecting multi-leaf diseases, shortly the LeafDoc-Net. Transfer learning is a highly effective approach in deep learning that offers several advantages over training models from scratch for new tasks. The method has gained substantial attention and acknowledgment owing to its accelerated training process, lower data requirement, robustness, adaptability, efficient feature extraction, and enhanced generalization capability. The proposed framework defines how plant leaf images are classified as healthy and diseased, followed by their utilization in identifying various diseases. The overall contribution of this work:

- Presents a novel architecture, “LeafDoc-Net”, which is a faster, robust, lightweight, precise, accurate, efficient multi-leaf disease detection framework based on DenseNet121 and MobileNetV2 integrating effective data augmentation, preprocessing, concatenation, attention-based transition, batch normalization, global average pooling, and more dense layers with swish activation
- Suggests a range of optimization techniques to effectively tackle the challenges of underfitting and overfitting in complex and less amount of image datasets to achieve the highest possible performance
- Includes the comparison result between the proposed architecture and the experimented state-of-the-art models in terms of every performance metric, including accuracy, precision, recall, and AUC, for the cassava and wheat Leaf disease dataset
- Describes model interpretability through the Grad-CAM++, incorporated into the proposed architecture

The rest of the paper is organized as the related works in section 2, the material and methods in section 3, the result analysis in section 4, the discussion in section 5, and the conclusion and the future work in section 6 followed by the references.

## 2 Related works

Different approaches, including K-means clustering, SVM (Islam et al., 2017), KNN (Singh and Kaur, 2019), and ANN classifier (Dhakate and Ingole, 2015), have been deployed to identify plant diseases and classification. Nevertheless, there is a need to enhance the practical outcomes of these algorithms and other algorithms used in image processing. These approaches also consume significant time when applied to disease classification (Narayanan et al., 2022). Certain plant diseases lack well-defined boundaries and can instead blend with healthy leaf tissue, posing a challenge for their identification using current techniques. Therefore, developing a comprehensive classification system is imperative to overcome these limitations effectively. In order to tackle these challenges, the agricultural sector has employed advanced DL algorithms. A recent research effort has demonstrated that DenseNet-121 and other contemporary pre-trained models, including VGG-16, ResNet-50, and Inception-V4, successfully detected and classified plant diseases. The model demonstrated a remarkable classification accuracy of 99.81% when evaluated on the Plant Village dataset, outperforming other models in performance (Eunice et al., 2022).

Transfer Learning is currently considered the most advanced technique for improving the performance of CNN-based classifiers in plant leaf disease detection. Transfer learning strategies utilize the knowledge and skills obtained from prior tasks to tackle the current problem effectively. It has become very popular in deep learning due to its notable performance in scenarios lacking data. Krishnamoorthy et al. (2021) conducted a study in which they demonstrated the application of a pre-trained DCNN InceptionResNetV2 with a transfer learning approach. They incorporated different fine-tuning hyperparameters and achieved a final accuracy of 95.67%. In a separate investigation conducted by Latif et al. (2022), the primary objective was to develop a robust methodology for precisely identifying and diagnosing healthy leaves and five distinct diseases. This was achieved by employing a transfer learning algorithm based on the VGG19 architecture. The modified technique demonstrated the highest average accuracy of 96.08%. The model’s accuracy yielded precision, recall, specificity, and F1-score values of 0.9620, 0.9617, 0.9921, and 0.9616, respectively. Simhadri and Kondaveeti (2023) utilized the transfer learning technique to diagnose and efficiently manage rice plant diseases. This involved utilizing a collection of 15 pre-trained CNN models. The results indicated that the InceptionV3 model performed better than others, attaining a notable average accuracy rate of 99.64%. The Precision, Recall, F1-Score, and Specificity values for InceptionV3 were documented as 98.23%, 98.21%, 98.20%, and 99.80%, respectively. In contrast to the other models investigated in the study, the AlexNet model demonstrated relatively lower performance, achieving an average accuracy of 97.35%. In an attempt to significantly enhance the accuracy of maize leaf disease detection, Rajeena PP et al. (2023) suggested an architecture that involves adjusting the variables of EfficientNet. The authors asserted that their suggested approach achieved recognition accuracy of 98.85%, notably superior to other state-of-the-art techniques. In their study, Mukti and Biswas (2019) introduced a plant disease detection model that utilizes transfer learning, focusing on employing the ResNet50 CNN architecture. Their study mainly emphasized utilizing the ResNet50 network as the pre-trained model in the transfer learning technique. Wu et al. (2022b) presented an enhanced model utilizing ResNet101 to identify diseases in woody fruit plant leaves. This study utilized a dataset of six distinct types of fruits affected by a leaf disease. The dataset was classified into 25 categories according to species, disease type, and severity level. To address the overfitting issue, the authors implemented several techniques, such as global average pooling, layer normalization, dropout, and L2 regularization. Furthermore, the researchers integrated the Squeeze-and-Excitation Network (SENet) attention mechanism into the model to augment its feature extraction capabilities. The outcomes from the research indicate that the suggested architecture exhibited an overall accuracy rate of 85.90% in effectively classifying leaf diseases in woody fruit plants. Ferentinos (2018) proposed a CNN architecture for disease detection and classification across a diverse range of 25 plant species. Among the CNN models that were evaluated, it was found that the VGG architecture exhibited the highest level of performance, achieving an accuracy rate of 99.48% on the given dataset. Similarly, Mohanty et al. (2016) introduced a method based on deep learning, which involved training a dataset comprising 54,000 images. This dataset encompassed 14 distinct crop species and 26 classes representing disease and healthy conditions. Among the various models that were tested, GoogleNet with transfer learning exhibited the highest level of accuracy, reaching 99.34%.

The popularity of advanced deep learning algorithms, particularly transfer learning, has extended to detecting and classifying leaf diseases in cassava and wheat. In order to detect cassava leaf diseases, Sambasival et al. (Sambasivam and Opiyo, 2021) proposed a cassava disease detection approach demonstrating high efficiency in identifying various cassava leaf diseases. This method was developed and evaluated using a dataset of 10,000 images collected from Uganda. The accuracy of the proposed method surpassed 93% through the utilization of deep CNNs. In practical implementations of disease detection in field settings, it is imperative to consider the limitations imposed by low-resolution capturing devices. Hence, Abayomi-Alli et al. (2021) conducted an experiment in which deliberate destructive filters were employed to reduce the quality of the images in the Cassava disease dataset. Subsequently, a deep network was trained using MobileNetV2 as a foundation for this experiment. In their study, Ramcharan et al. (2017) employed transfer learning techniques to train a deep CNN to detect multiple diseases and pests affecting cassava plants. The researchers achieved an impressive overall accuracy rate of 93% by applying a dataset collected from various fields in Tanzania. Chen et al. (2021) introduced a novel CNN architecture named Efficientnet in their study. This framework demonstrates the ability to classify various cassava leaf diseases by utilizing low-bandwidth images. The proposed model achieved an accuracy of 89% when evaluated on five distinct cassava leaf datasets. Goyal et al. (2021) proposed a deep CNN architecture for identifying and categorizing wheat leaf diseases. The model achieved an accuracy rate 97.88% in detecting and classifying ten different types of leaf and spike wheat diseases. In a study, Mi et al. (2020) introduced an automated approach using the DenseNet architecture to assess the severity grading of wheat rust disease. The proposed method was evaluated on the wheat stripe rust grading dataset (WSRgrading dataset). The results demonstrated an accuracy of 92.53% achieved by the model. Xu et al. (2023) introduced a novel RFE-CNN approach for detecting wheat leaf diseases in their study. This method demonstrated superior performance to popularly employed CNN architectures such as VGG, Inception, and EfficientNet. The RFE-CNN achieved an average classification accuracy of 98.83%. Fang et al. (2023) proposed a novel lightweight multiscale CNN model that incorporates techniques to enhance attention toward disease portions of images while reducing attention toward complex backgrounds. This method’s results indicate a high accuracy level, specifically 98.7% when applied to a dataset of seven distinct classes. In their study, Jiang et al. (2021) researched to enhance the performance of the VGG16 model, which had been pre-trained on the IMAGENET dataset. The authors focused on fine-tuning this model using images of rice and wheat leaf diseases. Notably, their approach achieved an impressive accuracy rate of 98.75%, specifically for wheat leaf diseases. Genaev et al. (2021) introduced an alternative approach to transfer learning, which focuses on automating wheat fungi disease identification. Their method utilizes the EfficientNet architecture and achieves a notable accuracy of 94.2% on a dataset of 2414 instances of wheat fungi disease. Notably, 86% of the images in this dataset were labeled as having either a single or multiple diseases.

Many researchers attempted to find lightweight architectures for leaf disease detection for low-resource computational devices. Arun and Umamaheswari (2023) presented a lightweight architecture using the Complete Concatenated Deep Learning (CCDL) architecture to classify agricultural diseases across crops. The Complete Concatenated Block (CCB) manages model parameter count with a point convolution layer before each convolution layer. The architecture is trained on the Plant Village dataset. A Pruned Complete Concatenated Deep Learning model is created after training. The study includes three architecture options, with PCCDL-PSCT standing out. This version classifies 98.14% with a 10-megabyte model. In their study, Yang et al. (2023) introduced DGLNet, a rice disease diagnosis network that is both lightweight and accurate. The Global Attention Module (GAM) and Dynamic Representation Module (DRM) are modules inside the DGLNet framework that have a modest level of complexity. The GAM effectively captures essential information in intricate and noisy environments, enhancing the model’s generalization capabilities. The DRM system has devised a proprietary technique known as the four-dimensional flexible convolution (4D-FConv), which effectively creates adaptive convolutional kernel parameters by utilizing four dimensions. The proposed methodology demonstrates superior performance compared to widely used approaches, achieving recognition accuracies of 99.82% and 99.71% on two authentic plant disease datasets. The study conducted by Gehlot and Gandhi (2023) involved the utilization of an enhanced, efficient, and tailored Deep Convolutional Neural Network (DCNN) on the plant village dataset. The primary objective of this study was to discern various diseases affecting tomato plants. This study aims to conduct a comparative analysis and practical implementation of MobileNet and a tailored lightweight model derived from MobileNetV2 for picture categorization. The dataset has 14,529 images depicting tomato leaves, categorized into ten distinct classes. The proposed model demonstrates high accuracy, with a success rate of 99.26%. Sharma et al. (2023) introduced DLMC-Net, a more profound lightweight convolutional neural network architecture, for real-time plant leaf disease detection across multiple crops. The suggested model extracts deep features using a succession of collective blocks and the passage layer. This helps propagate and reuse features, solving the vanishing gradient problem. Point-wise and separable convolution blocks are used to reduce trainable parameters. The DLMC-Net model is tested on four public datasets: citrus, cucumber, grapes, and tomato. The experimental results of the proposed model were compared to seven state-of-the-art models. In experiments with citrus, cucumber, grapes, and tomato, the suggested model outperformed all other models with an accuracy of 93.56%, 92.34%, 99.50%, and 96.56%, respectively, under difficult background conditions. Fan and Guan (2023) developed VGNet, a corn disease recognition system based on pre-trained VGG16, with batch normalization (BN), global average pooling (GAP), and L2 normalization. Transfer learning for corn disease categorization improves the proposed strategy. The Adam optimizer outperforms SGD for agricultural disease identification in experiments. The model achieves 98.3% accuracy and 0.035 loss at 0.001 learning rate. Nine corn diseases have precision and recall values between 98.1% and 100% after data augmentation. Zhong et al. (2023) developed LightMixer, a lightweight tomato leaf disease diagnosis model. The LightMixer model uses Phish and light residual modules for depth convolution. Phish is a lightweight convolution module that employs depth convolution to splice nonlinear activation functions and lightweight convolutional feature extraction for deep feature fusion. Lightweight residual blocks were used to build the light residual module to increase network design computational efficiency and prevent disease feature information loss. LightMixer obtained 99.3% accuracy on public datasets with 1.5 M parameters in experiments. Guan et al. (2023) presented Dise-Efficient, a network architecture based on EfficientNetV2, to improve plant disease and pest diagnosis. Their findings show that training this model with dynamic learning rate degradation improves plant disease and pest recognition. Transfer learning improves the model’s generalization capacity during training. Experimental data showed that the model achieves 99.80% accuracy on the Plant Village plant disease and pest dataset. Transfer learning on the IP102 dataset, which simulates real-world environmental circumstances, gives the Dise-Efficient model 64.40% plant disease and pest recognition accuracy.

The majority of the existing research has primarily concentrated on positive performance outcomes. While previous research has demonstrated impressive results, several areas for improvement still need to be addressed, including optimizing model complexity and robustness. Moreover, the absence of other significant performance metrics is evident. To effectively address the issue of insufficient complex data, it is imperative to conduct careful experiments. The present research necessitates a comprehensive examination of frequent issues, including complex, less research data, underfitting, and overfitting. Moreover, most of the research in the literature utilized a leaf segmentation method, which is costly in terms of resources and time. This research presents a solution to the various challenges associated with plant disease without leaf segmentation, making the proposed framework cost-effective, fast, and efficient for farmers. It involves the development of a robust and lightweight architecture for detecting leaf diseases in complex real-time scenarios for multiple plant species. Moreover, the proposed architecture also introduces a state-of-the-art technique to mitigate complex, less research data, underfitting, and overfitting issues.

## 3 Materials and methods

### 3.1 Dataset

**Figure 1:**
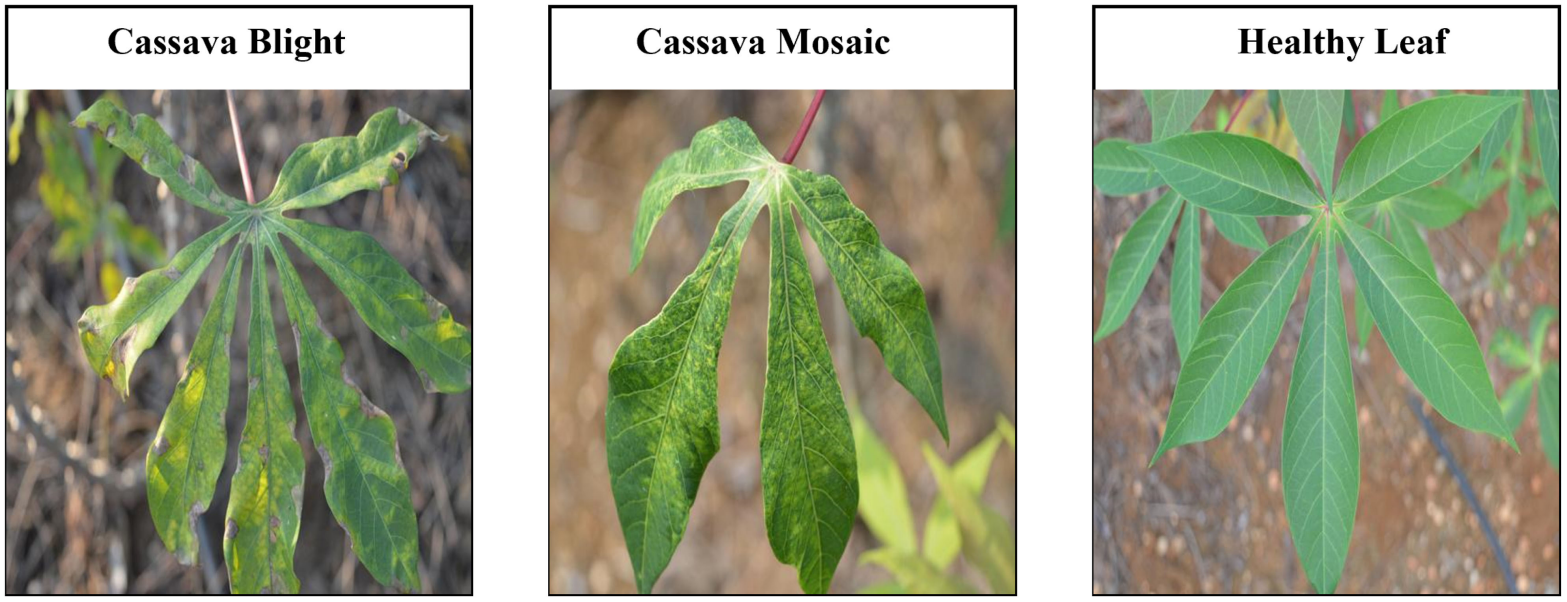
Sample image per class of cassava leaf disease dataset.

**Figure 2:**
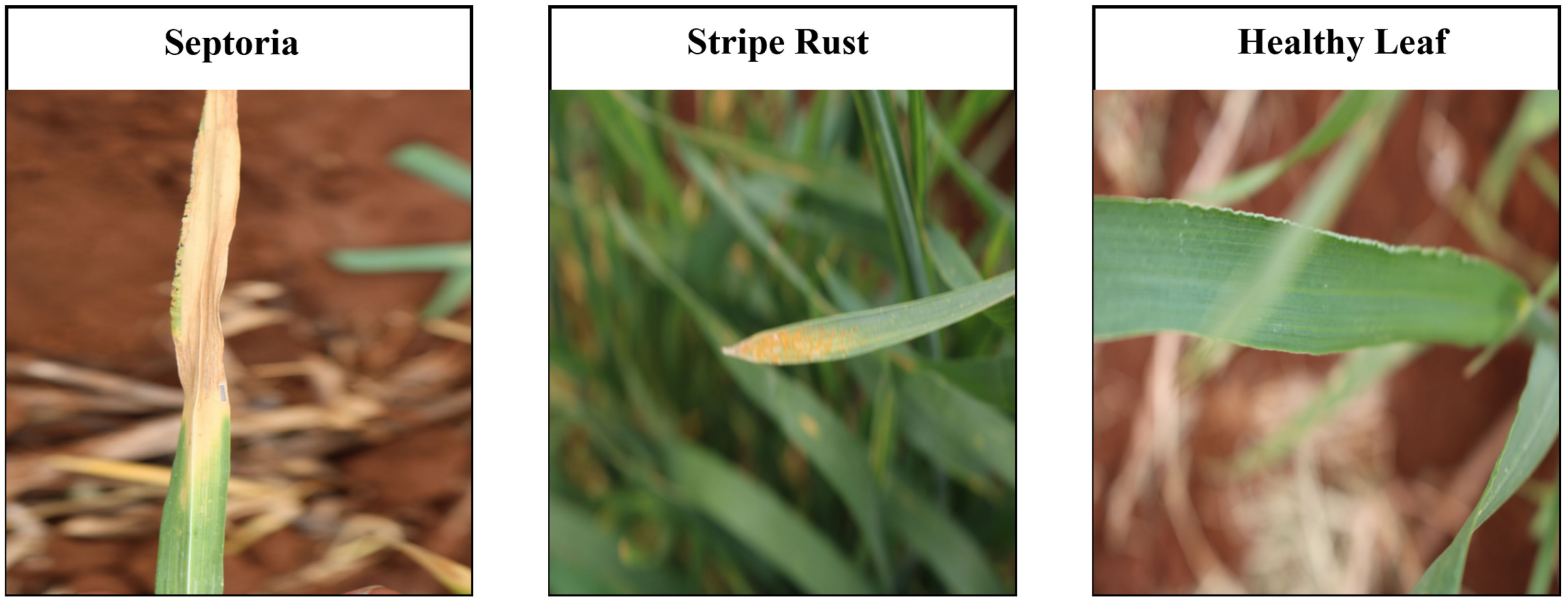
Sample image per class of wheat leaf disease dataset.

An appropriate dataset is required at each stage of the research process, starting from training and extending to the evaluation of algorithm effectiveness. Since our main goal was to develop a lightweight, robust, and accurate leaf disease detection system regardless of leaf size from fewer images and complex backgrounds, we selected two datasets: cassava leaf disease (L G et al., 2021), and wheat leaf disease dataset (Hawi, 2021) which matched all our selection criteria. All the datasets comprise two disease classes and a class representing healthy samples. These datasets have significantly fewer images per class. The datasets are publicly available on Mendeley. We split every dataset’s image into two groups: train (80%) and test (20%). **Table 1** shows detailed information on the datasets, train, and test images per class. **Figures 1**, **2** represent the sample image per class of cassava and wheat leaf disease dataset.

**Table 1:** The number of images depicting leaf diseases across various datasets.

Dataset	Class	Train	Test
Cassava Leaf Disease	Cassava CB (Cassava Blight)	39	10
	Cassava CM (Cassava Mosaic)	70	18
	Cassava Healthy leaf	73	18
Wheat Leaf Disease	Healthy	81	21
	Septoria	78	19
	Stripe Rust	169	39

### 3.2 Data augmentation and preprocessing

A substantial quantity of data is imperative for the deep learning algorithm to identify and classify leaf diseases accurately (Li et al., 2021a). Generating extra data from existing data is called “data augmentation”. Furthermore, this approach could address the problem of overfitting commonly occurring in CNN training. Thanks to data augmentation approaches, a more extensive dataset can be employed during training, which increases generalization without endangering overfitting (Jiang et al., 2019). We enhanced the leaf images to mimic actual conditions by shearing, rotating, adjusting the height and width, and flipping them horizontally. The dataset images went through preprocessing before being used to train the model to increase feature extraction and classifier performance. The RGB coefficients in the dataset used in this study ranged from 0 to 255. Processing images with higher values, nevertheless, takes much work. To overcome this situation, all of the images in the dataset were normalized using a scale factor of 1/255, yielding values between 0 and 1. The dataset’s data has been standardized to a fixed pixel size of 224*224 to facilitate training. Utilizing neural networks with higher-resolution images would require four times as many input pixels, increasing training time (Saponara and Elhanashi, 2021).

### 3.3 Experimental setup

We trained various pre-trained models using the Nvidia GeForce GTX 1650 Max-Q GPU (4 GB), Keras, and TensorFlow libraries. This study investigated multiple factors: the number of images per batch, various epochs, augmentation settings, and activation functions. The models were resized by determining the appropriate input shape according to the specifications of each model. Effective augmentation methods were used, including rotation, vertical flipping, width shifting, and height shifting. All experiments utilized the identical optimizer, Adam, with a batch size 8. The learning rate was set to 1 × 10^−5^, and the number of epochs was fixed at 50, with early stopping implemented. Accuracy, precision, recall, and area under the curve (AUC) were included in the performance metrics.

### 3.4 The proposed “LeafDoc-Net” architecture

Several pre-trained models such as InceptionV3, ResNet152V2, InceptionResNetV2, MobileNet, MobileNetV2, NASNetLarge, DenseNet121, DenseNet169, DenseNet201, EfficientNetV2S, and EfficientNetV2L were employed to measure the detection performance on the cassava and wheat leaf diseases datasets. All the pre-trained models were trained without freezing any layer. With no learning process restrictions, this method helped the models comprehend the new challenge better. DenseNet201 performed better than other base models on the cassava leaf disease datasets. However, EfficientNetV2s outperformed DenseNet201 on the wheat leaf disease dataset. It proved that no classification model could perform similarly on all leaf disease datasets. Some pre-trained models encountered a significant issue of underfitting, representing significant limitations of the base models. Several Transfer-learning-based strategies, such as integrating various types of layers with the model, have been investigated to address the underfitting and overfitting issues. Transfer learning-based techniques dramatically enhance the detection performance of the proposed LeafDoc-Net architecture.

Before beginning the training process of the LeafDoc-Net architecture, a range of data augmentation and preprocessing techniques were employed to boost the architecture’s performance and address the threat of overfitting. We employed DenseNet121 and MobileNetV2 as the foundation of the LeafDoc-Net architecture. DenseNet121 was chosen for its parameter efficiency and dense connectivity, facilitating feature reuse and effective learning, while MobileNetV2 was selected for its efficiency and speed, making it suitable for resource-constrained environments. The combination of these models leverages DenseNet’s rich feature extraction capabilities and MobileNetV2’s computational efficiency, creating a hybrid architecture that excels in applications requiring detailed feature extraction and adaptability to limited computational resources. The ensemble effect and complementary strengths of each model enhance the overall performance of the proposed architecture. DenseNet121 is a convolutional neural network (CNN) architecture in the DenseNets (Densely Connected Convolutional Networks) family. DenseNets are designed to address some of the shortcomings of traditional CNN architectures, such as VGG and ResNet, by encouraging feature reuse, addressing the vanishing gradient issue, and improving model compactness. The DenseNet121 design is built around several dense blocks. Each dense block is made up of multiple closely connected convolutional layers. Each layer in a dense block takes input from the layers above it and gives its feature maps to the layers below it. This high degree of connectivity increases feature reuse and gradient flow during training (Huang et al., 2017). The Mathematical equation for the dense block in Equation 1 is as follows:


(1) $$x_{1}=H_{l}\left(\left[x_{0},x_{1},\ldots ,x_{l-1}\right]\right)$$

Where, $\left[x_{0},x_{1},\ldots ,x_{l-1}\right]$ denotes the result of the concatenation of all of the feature-maps created in layers 0,1,. . .,*l* − 1, *H_l_* has been described as a composite function that consists of three sequential operations on the *l* th layer.

**Figure 3:**
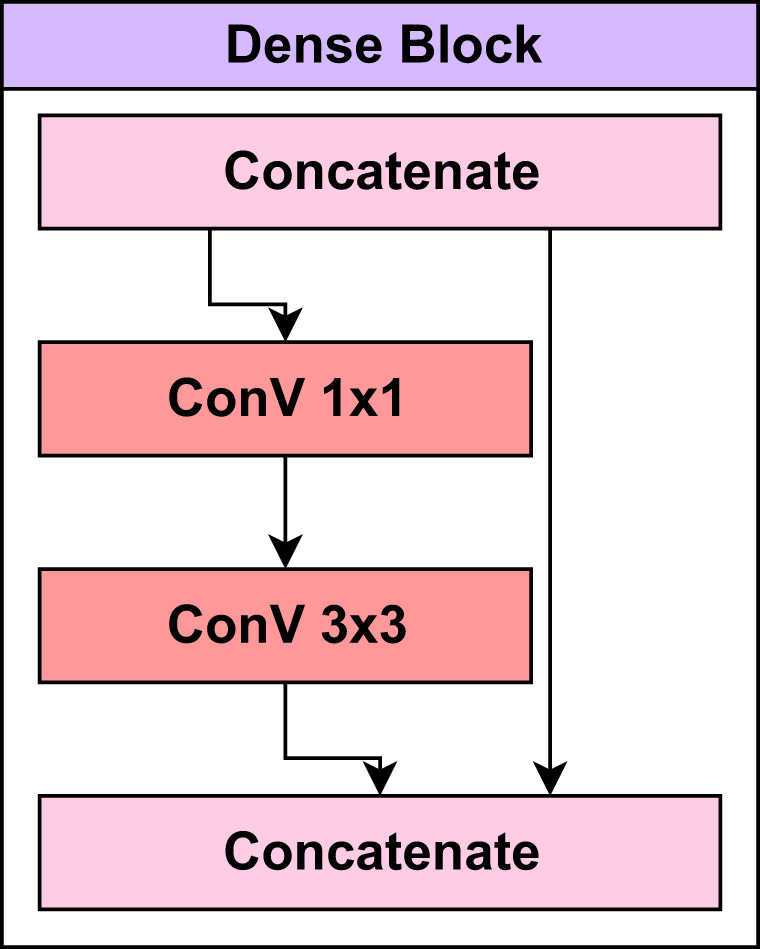
The layers in the dense block of the pre-trained DenseNet121 model.

**Figure 3** presents the layers in the dense block of the pre-trained DenseNet121 model.

Transition layers between dense blocks reduce the spatial dimensions of the feature maps and the number of channels. This aids in reducing computational complexity and managing the model’s growth. Convolutional, batch normalization and pooling layers are standard components of transition layers. Within each dense block, there are bottleneck levels. 1x1 convolutional layers are utilized before the standard 3x3 convolution to reduce the number of channels. This reduction in channel count contributes to a reduction in computing complexity (Huang et al., 2017). The mathematical equation presented in Equation 2 for the transition layer can be defined as:


(2) $$z^{l}=W^{l}\times f_{1}\left(z^{l-1}\right)+b^{l}$$

Where, *z^l^* is the *l^th^* layer neuron status, *f*_1_() is the activation function. *W^l^* and *b^l^* are the weight matrix and bias from (*l* − 1)*^th^* to the *l^th^*, respectively.

**Figure 4:**
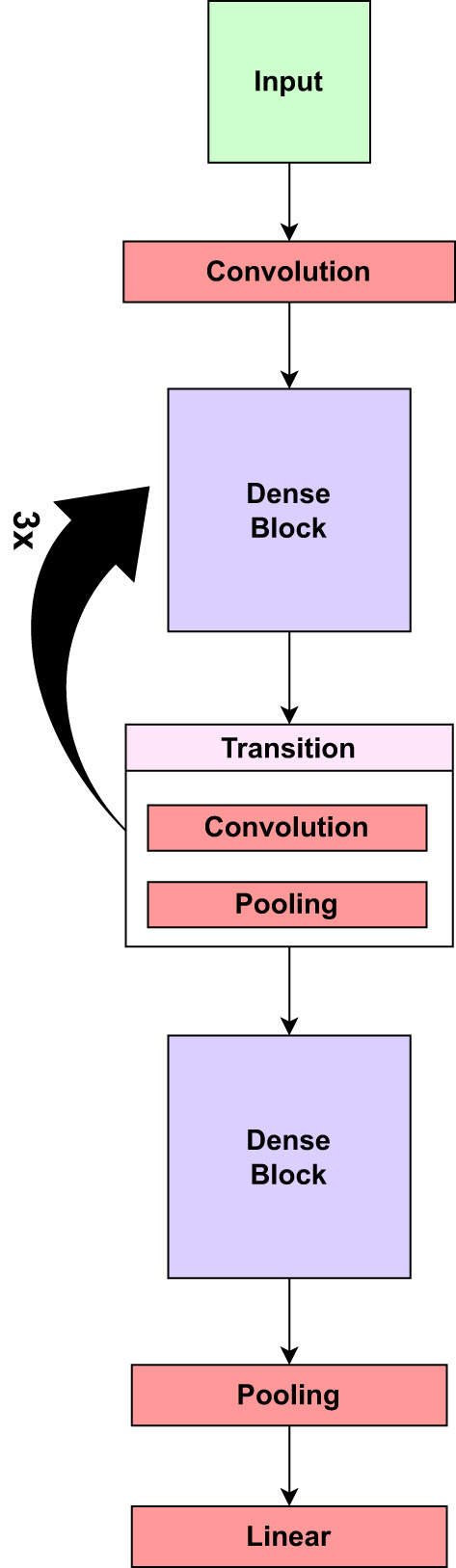
The core components of the pre-trained DenseNet121 model.

DenseNet121 is a deep network with fewer parameters than previous deep architectures, allowing it to be trained on standard hardware. **Figure 4** presents the core components of the pre-trained DenseNet121 model.

**Figure 5:**
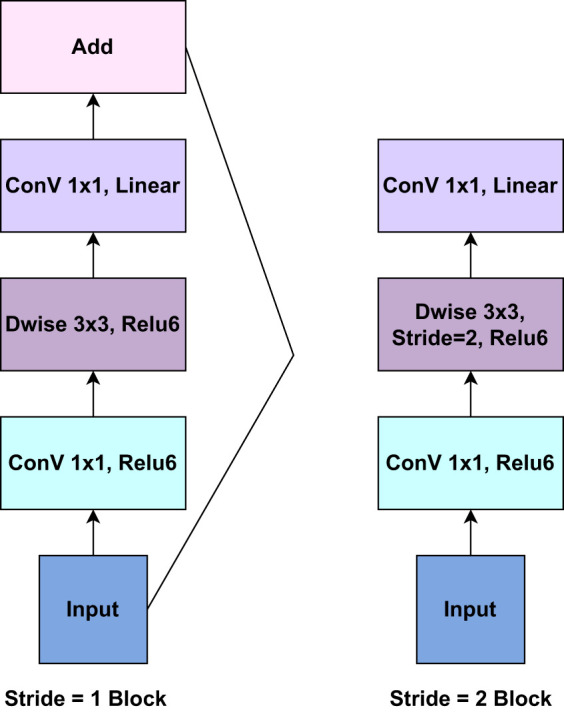
The core components of the inverted residuals layer of the pre-trained MobileNetV2 model.

On the other hand, MobileNetV2 is a convolutional neural network (CNN) architecture that is optimized for on-device vision applications. It enhances the original MobileNet architecture to improve accuracy and efficiency. The concept of “inverted residuals” is introduced in MobileNetV2 to make the network deeper while keeping computing costs low. Each inverted residual block typically has an Expansion Layer (1x1 convolution), Depthwise Separable Convolution layer, Projection Layer (1x1 convolution), and Skip Connection layer. The expansion layer (1x1 convolution) increases the channels, allowing the model to capture more complicated features. The depthwise separable convolution is the foundation of MobileNetV2. It is divided into two significant steps: Depthwise Convolution and Pointwise Convolution. Each input channel is convolved separately with its associated filter in Depthwise Convolution, resulting in a set of feature maps. Following the depthwise convolution, the feature maps from the preceding phase are combined using a 1x1 convolution (pointwise convolution). This contributes to the model’s depth (number of channels) and capacity. The projection layer decreases the number of channels to a more manageable number while keeping essential information and lowering computational complexity. A skip connection (or residual connection) is added to the projection layer’s output, which allows gradients to flow freely and prevents the vanishing gradient problem. MobileNetV2 also employs bottleneck blocks with 1x1 and 3x3 convolutions to reduce computing costs further. Inverted Bottlenecks are inverted residual block extensions. They boost network depth and are employed at deeper levels. Linear bottlenecks are used to reduce the number of channels while avoiding the addition of non-linearity. MobileNetV2, like other recent CNN architectures, uses Global Average Pooling at the end to turn spatial input into a fixed-size vector for classification (Sandler et al., 2018). **Figure 5** presents the core components of the inverted residuals layer of the pre-trained MobileNetV2 model.

In order to enhance the ability of the proposed architecture, we incorporated an attentive transition layer comprising 1024 filters with the DenseNet121 model. The process of attentive transition begins with computing the attention map through the global average pooling of input feature maps, reducing their spatial dimensions to a single value per channel. This attention map is then processed using a dense layer with 1024 filters and a sigmoid activation function, learning attention weights for input channels. Next, the attention map is reshaped to (1, 1, 1024 filters) for element-wise multiplication with the input feature maps, enhancing or diminishing specific characteristics based on learned attention weights. Following this attention application, a convolutional layer with 1024 filters and a 1x1 kernel size further refines the feature maps. Subsequently, spatial dimensions are reduced using max pooling with a 2x2 pool size and stride.

The attentive transition module’s role is to compute attention weights for different channels in the input feature maps, allowing the model to emphasize essential features and prioritize discriminative information selectively. This adaptive refinement enhances meaningful representations by boosting crucial features and suppressing noise. The attentive transition enhances computational efficiency while maintaining critical information by employing dimensionality reduction techniques like reducing the number of filters and max pooling. This approach captures higher-level spatial patterns and abstract representations while retaining spatial dependencies for improved class discrimination (Mi et al., 2020).

We also modified the MobileNetV2 model by adding an extra attention layer. The attention layer computes attention weights using a 1x1 convolutional layer followed by a sigmoid activation function, yielding attention values ranging from 0 to 1. These attention weights are subsequently applied to the original feature maps through element-wise multiplication. This technique emphasizes specific regions in feature maps based on their relevance as indicated by attention weights. The resulting attended features contain the original feature information but with a stronger emphasis on essential locations, making this method ideal for applications like object recognition or segmentation, where the model needs to choose to emphasize crucial image regions for better performance (Mi et al., 2020).

After modifying the DenseNet121 and MobileNetV2 models, we added a global average pooling layer with each model. In this study, the use of global average pooling was preferred over the implementation of a flattened layer. This choice was made to expedite the training process by reducing the parameters in the proposed architecture. Utilizing the global average pooling layer effectively addresses the problem of overfitting. Outputs from the global average pooling layer of each model fed into the concatenate layer. This concatenate layer concatenates feature maps from the DenseNet121 and MobileNetV2 models. The Concatenation of feature maps from two different models enables the proposed architecture to learn diverse representations of features from the input image. The concatenated feature maps can extract more patterns and information from an image. This can lead to better generalization, making the proposed architecture more robust and capable of managing many sorts of information. Concatenating feature maps helps the model discriminate between objects or similar classes. Distinct models may concentrate on distinct elements of an object, and their combination may provide more discriminative ability. Combining feature maps from different models can assist in reducing overfitting by adding redundancy and regularization. This is very helpful when working with limited training data (Pan et al., 2019).

Additionally, we implemented a dense layer consisting of 1024 filters with swish activation, followed by batch normalization, as it increases the reliability and quickness of our proposed architecture. Furthermore, we integrated another dense layer with 512 filters, utilizing swish activation and batch normalization, followed by an additional dense layer with 128 filters to increase the complexity of the proposed architecture. Including additional dense layers in the architecture boosts its capacity to extract supplementary features and improve overall performance (Josephine et al., 2021).

Our study conducted experiments by incorporating the Rectified Linear Unit (ReLU), Parametric Rectified Linear Unit (PReLU), and Swish activation functions with all the additional dense layers into our proposed architecture. Swish’s performance was superior to using ReLU and PReLU across all performance metrics. Ramachandran et al. (2017)’s experimentation showed that the Swish activation function outperformed ReLU on complicated datasets. The faster convergence of this smooth, non-monotonic function makes data normalization conceivable. The activation function swish is defined mathematically in Equation 3 as follows:


(3) f(x)=x−σ(x)

Where, *σ*(*x*) = (1 + exp(−*x*)) − 1) is the sigmoid function.

Compared to ReLU and PReLU, the Swish activation function is a promising new activation function with many advantages. It is more effective, expressive, and has a better gradient flow. It has also been demonstrated to perform better in practice on several tasks, including our experiment. Finally, we utilized a dense layer with an activation function called SoftMax (Sharma et al., 2017). Determining how many neurons are required in this layer was predicated upon the number of classes present within the dataset. The SoftMax function is frequently utilized when addressing multiclass classification tasks. The function generates a probability distribution across various classes, assigning each class a probability value ranging from 0 to 1. The SoftMax function assigns a higher probability to the target class than the remaining classes, indicating its likelihood as the predicted class. The activation function SoftMax is formally defined in Equation 4 as follows:


(4) $$softmax\left(z_{i}\right)=\frac{\text{exp }(z_{i})}{\sum_{i}\exp\;(z_{j})}$$

Where, *z* = values of the output layer’s neurons, and exp serves as the non-linear function.

We conducted experiments to determine the effects of deploying various learning rates for modifying hyperparameters in the Adam optimizer. In the proposed architecture, we subsequently employed the Adam optimizer with a learning rate of 1 × 10^−5^. The loss function employed in this study for the proposed architecture, which aims to address multiclass classification problems, is categorical crossentropy (Li et al., 2022). The mathematical equation of categorical crossentropy presented in Equation 5 as:


(5) $$L_{i}=-\sum_{j}t_{i,j}\text{log }(P_{i,j})$$

where, *P* = predictions, *t* = targets, *i* = data points, *j* = class.

**Figure 6:**
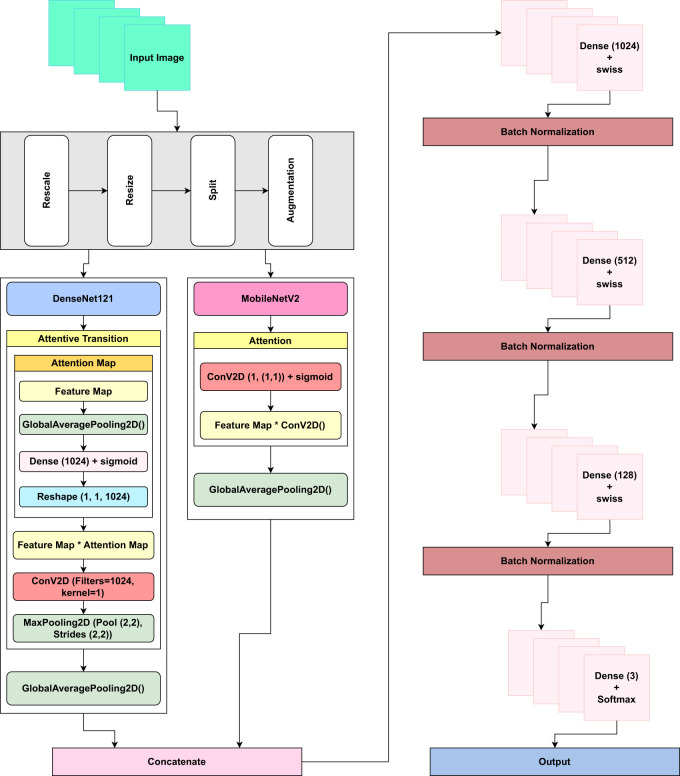
The figure shows the core elements of the proposed LeafDoc-Net architecture. The foundation models are DenseNet121 and MobileNetV2, with additional layers in DenseNet121 and MobileNetv2. Additional layers in the proposed LeafDoc-Net architecture include concatenating two models, several dense layers with swish activation, batch normalization, and the output layers with softmax activation.

Furthermore, to mitigate the problem of overfitting, an early stopping function was implemented. The function was configured by setting the monitoring parameter to ‘val loss’ and assigning a patience value of 3. Once the validation loss value exhibits an upward trend, signifying the possibility of overfitting, the model’s training process is automatically terminated. This measure aids in mitigating the model’s tendency to train continuously on data that could potentially decrease its overall ability to generalize. **Figure 6** illustrates the core elements of the proposed architecture.

### 3.5 Performance metrics

An essential part of developing a deep learning model is assessing its performance. Performance or assessment metrics assess a model’s performance on the provided data. Using these measurements, the researchers may better understand how closely the data and model match and adjust its parameters to increase effectiveness. Different evaluation measures have been used in this experiment to explain how well the model performed. Below is a brief explanation of the evaluation criteria utilized for the study.

#### 3.5.1 Confusion matrix

Confusion matrix (CM) is utilized as *NxN* arrays to evaluate the outcome of a deep learning-based algorithm, with *N* representing the total number of classes within the predefined set. This matrix facilitates the evaluation of the model’s predictions compared to the actual values. By utilizing a confusion matrix, researchers can comprehend the efficiency of their deployed algorithm and the various types of errors it may generate. The insights provided by these evaluations are of significant value as they enable researchers to analyze and enhance the model’s performance across various classes.

#### 3.5.2 Accuracy

Accuracy (AC) is a widely employed technique in deep learning to determine a classification algorithm’s ability. It provides a straightforward method for comprehending the model’s overall performance by calculating the proportion of correctly classified samples relative to the total number of samples within the dataset. Nevertheless, the usefulness of this metric may be limited, particularly in the context of imbalanced datasets. Accuracy can be mathematically expressed (Equation 6) as:


(6) $$AC=\frac{TP+TN}{TP+FP+TN+FN}$$

#### 3.5.3 Precision

Precision is a commonly employed benchmark in the field of deep learning. The model’s performance is evaluated by determining the percentage of accurate positive predictions, which refers to correctly identifying positive samples among all the positive predictions made by the model. It focuses on the accuracy of a model’s positive predictions, which is crucial when working with unbalanced datasets or when the cost of false positives is high. High precision means the model is good at correctly predicting the positive outcome, which lowers the incidence of false positives. PR can be mathematically explained (Equation 7) as:


(7) $$PR=\frac{TP}{TP+FP}$$

#### 3.5.4 Recall

Recall is an indicator of statistics employed to evaluate the predictive accuracy of a model. The calculation involves dividing the count of correctly predicted positive outcomes by the overall count of actual positive occurrences. In contrast to the accuracy metric, which solely evaluates the proportion of correctly predicted positive occurrences out of all anticipated positives, recall considers the number of positive instances the model failed to identify. The recall metric quantifies the proportion of true positive instances correctly identified by the model, indicating the model’s ability to capture positive predictions. The mathematical definition of RE presented in Equation 8 is:


(8) $$RE=\frac{TP}{TP+FN}$$

Here, *TP* = true positive, *TN* = true negative, *FP* = false positive, *FN* = false negative.

Furthermore, we measured the Area Under the Curve (AUC) value, a performance parameter for classifiers. The AUC is used to evaluate the discriminatory ability of a model in distinguishing between different occurrences. It is a scalar value that ranges between 0 and 1, where a higher AUC signifies greater accuracy in the model’s predictions.

### 3.6 Interpreting with XAI

For complex systems that rely on neural networks, it is crucial to establish trust by explaining how and what the models predict. To establish sufficient confidence and reliance on the model, we employed Grad-CAM++ (Chattopadhay et al., 2018), an enhanced iteration of Grad-CAM, to elucidate our approach visually. It is a technique for visual explanation based on CAM.

#### 3.6.1 Grad-CAM++

The Grad-CAM technique applies to localizing multiple instances belonging to the same class. Moreover, the localization of the heatmap produced by Grad-CAM may demonstrate enhanced precision in accurately identifying the precise region associated with a particular class within an image. The Convolutional Neural Network (CNN) model’s predictions can be effectively visualized by employing Grad-CAM++ (Chattopadhay et al., 2018) to enhance the object localization procedure and depict the visual representation of multiple object occurrences within an individual image. The Grad-CAM++ technique employs the weighted mean of the positive portion of the partial derivatives of the feature maps derived from the ultimate convolutional layer. The mathematical formulation of Grad-CAM++ can be represented (Equation 9) as follows:


(9) $$W_{k}^{c}=\sum_{i}\sum_{j}\alpha_{i,j}^{kc}ReLU\left(\frac{\partial Y^{c}}{\partial A_{i,j}^{k}}\right)$$

where, $W_{k}^{c}$ = neuron weights, $\alpha_{i,j}^{kc}$ = signifies the importance of location (*i,j*), *A^k^* = activation map, *c* = target class, *Y^c^* = The score of a network’s class *c*.

The effective use of pixel-wise ReLU on the final activation map is of utmost importance as it amplifies the characteristics that positively influence the desired class.

## 4 Result and analysis

**Figure 7:**
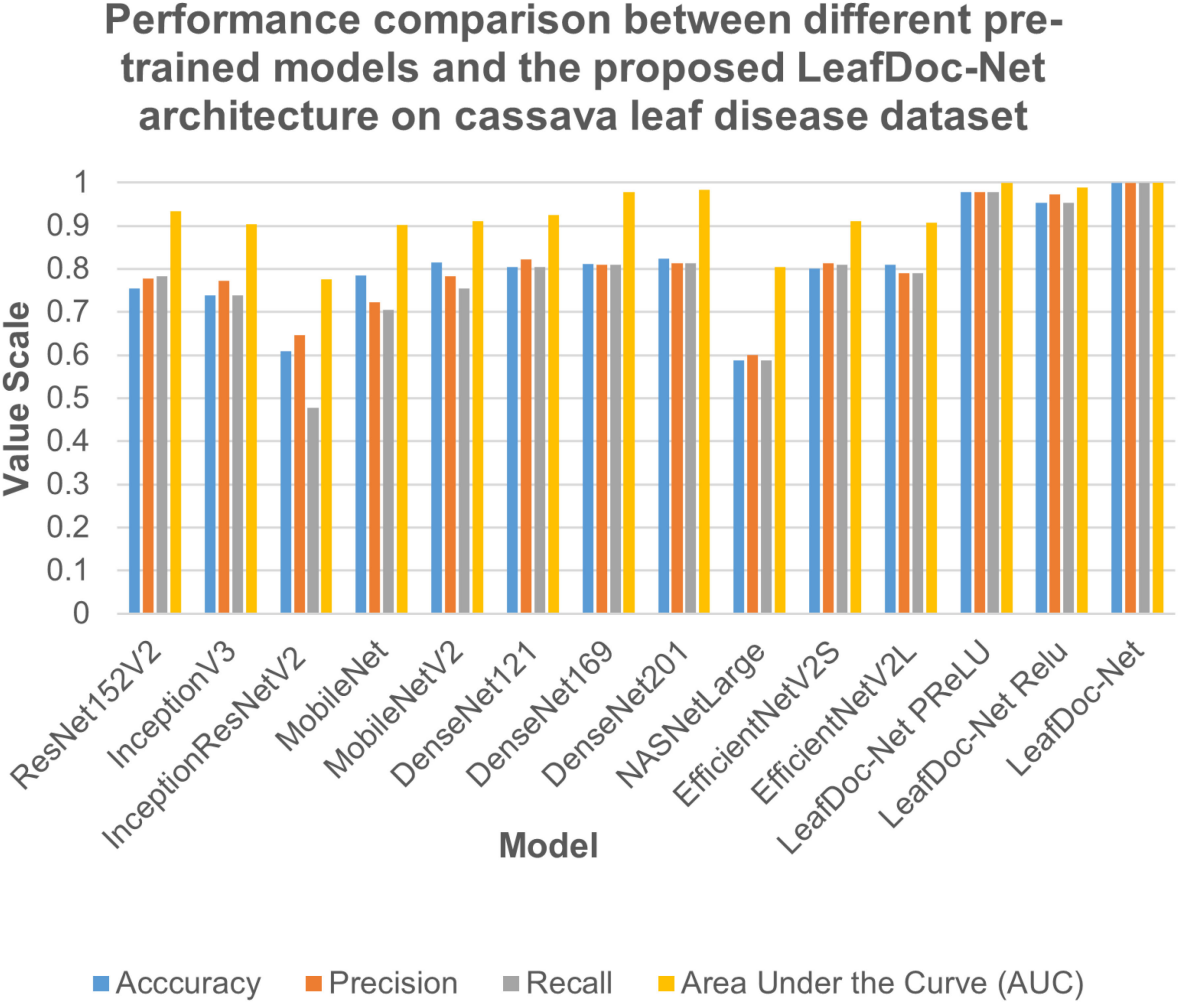
Performance comparison between different pre-trained models and our proposed LeafDoc-Net architecture on cassava leaf disease dataset.

**Figure 8:**
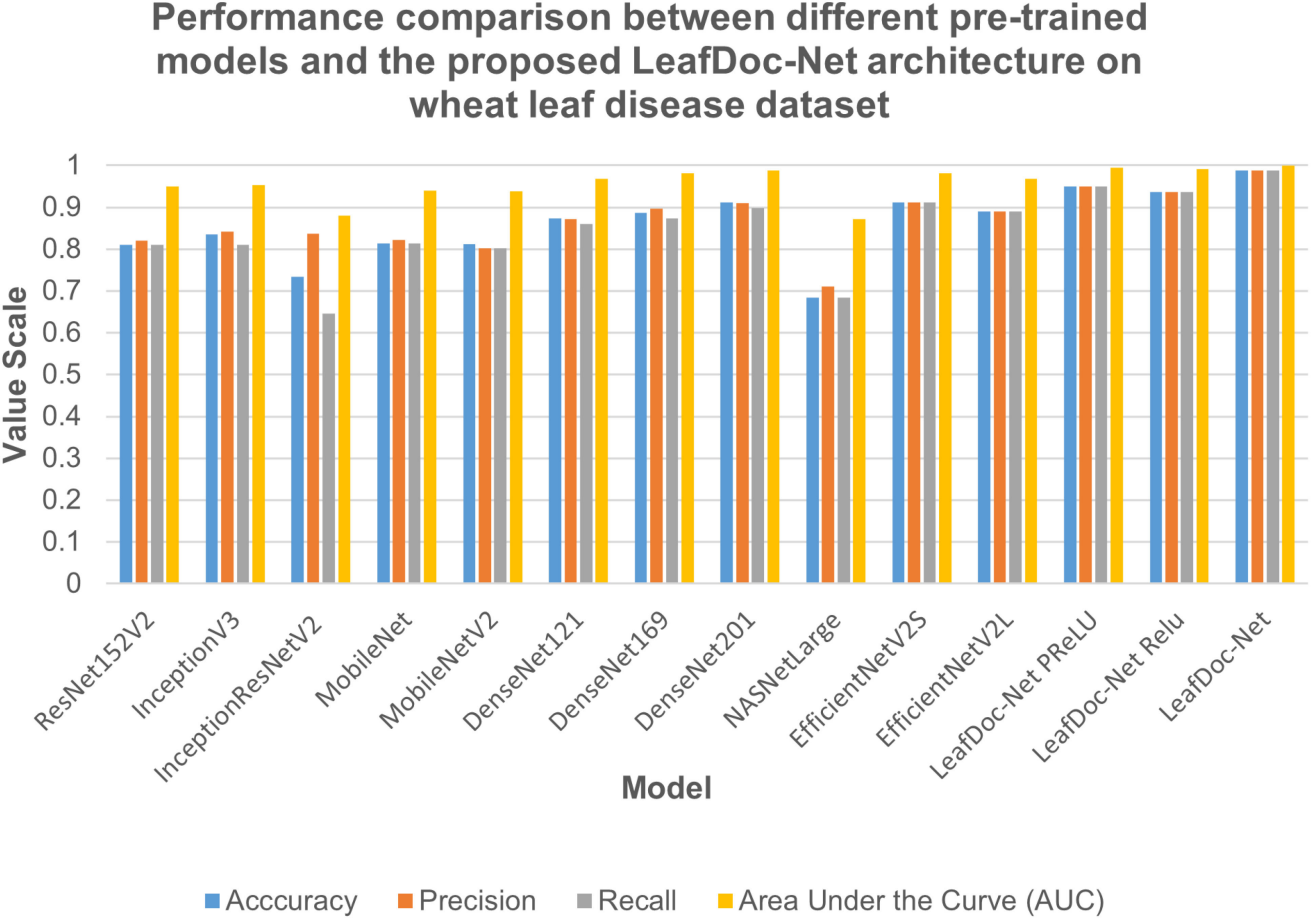
Performance comparison between different pre-trained models and our proposed LeafDoc-Net architecture on wheat leaf disease dataset.

Precise diagnosis is crucial for cases involving plants that directly affect human beings. This study proposes a transfer learning-based architecture called LeafDoc-Net to construct a lightweight and robust deep-learning model. We utilized two distinct datasets, cassava and wheat Leaf Disease, to compare the proposed and existing CNN pre-trained models. The objective was to assess the weight and robustness of the LeafDoc-Net architecture and compare it with the existing CNN model. The LeafDoc-Net architecture was analyzed by evaluating various performance metrics, including accuracy, precision, recall, and area under the curve (AUC) value. Other important factors, such as loss and total parameters, were also analyzed. The DenseNet201 pre-trained model outperformed all other pre-trained models on the cassava leaf disease dataset. However, on the wheat leaf disease dataset, EfficientNetV2S demonstrated superior performance compared to DenseNet201 and other pre-trained models. The pre-trained models showed issues of underfitting across the two leaf disease datasets, indicating a need for more robustness in their performance. The pre-trained models could not perform better due to the need for sufficient images per class in the selected two datasets. In deep learning, a large amount of input data is required to learn essential features and perform better. In order to solve this problem, we modified the DenseNet121 and MobileNetV2 pre-trained models by adding more complex layers to these models and combining them, resulting in satisfactory outcomes. After conducting various experiments involving modifications and hyperparameter tuning, the proposed LeafDoc-Net architecture showed significant superiority over all other pre-trained models. Our proposed architecture, LeafDoc-Net, displayed outstanding results compared to all the pre-trained models tested across the two datasets, resulting in the highest accuracy, precision, recall, and area under the curve (AUC) metrics. LeafDoc-Net achieved an accuracy score of 0.9999, a precision score of 0.9999, a recall of 0.9999, and an area under the curve (AUC) of 1.000 on the cassava leaf disease dataset. On the wheat leaf disease dataset, it performed similarly and achieved an accuracy of 0.9873, a precision of 0.9873, a recall of 0.9873, and an area under the curve (AUC) of 0.9996. **Tables 2**, **3** present comprehensive data regarding the performance of the proposed LeafDoc-Net architecture and various pre-trained models on the cassava and wheat disease datasets below. **Figures 7**, **8** visualize the comparison of the performance of different pre-trained models and our proposed architecture LeafDoc-Net.

**Table 2:** The performance summarization of different pre-trained models and proposed LeafDoc-Net architecture on cassava leaf disease dataset.

Model	Accuracy	Precision	Recall	Area Under the Curve (AUC)
ResNet152V2	0.7543	0.7771	0.7826	0.9348
InceptionV3	0.7391	0.7727	0.7391	0.9030
InceptionResNetV2	0.6087	0.6471	0.4783	0.7765
MobileNet	0.7846	0.7222	0.7043	0.9016
MobileNetV2	0.8143	0.7822	0.7543	0.9100
DenseNet121	0.8043	0.8222	0.8043	0.9257
DenseNet169	0.8113	0.8101	0.8096	0.9784
DenseNet201	0.8230	0.8130	0.8130	0.9831
NASNetLarge	0.5870	0.6000	0.5870	0.8040
EfficientNetV2S	0.8013	0.8124	0.8096	0.9108
EfficientNetV2L	0.8090	0.7910	0.7895	0.9080
LeafDoc-Net_PReLU	0.9783	0.9783	0.9783	0.9998
LeafDoc-Net_Relu	0.9530	0.9733	0.9530	0.9897
**LeafDoc-Net**	**0.9999**	**0.9999**	**0.9999**	**1.0000**

**Table 3:** The performance summarization of different pre-trained models and proposed LeafDoc-Net architecture on wheat leaf disease dataset.

Model	Accuracy	Precision	Recall	Area Under the Curve (AUC)
ResNet152V2	0.8101	0.8205	0.8101	0.9499
InceptionV3	0.8354	0.8421	0.8101	0.9533
InceptionResNetV2	0.7342	0.8361	0.6456	0.8802
MobileNet	0.8143	0.8212	0.8143	0.9407
MobileNetV2	0.8114	0.8014	0.8014	0.9386
DenseNet121	0.8734	0.8718	0.8608	0.9678
DenseNet169	0.8861	0.8961	0.8734	0.9807
DenseNet201	0.9114	0.9103	0.8987	0.9887
NASNetLarge	0.6835	0.7105	0.6835	0.8717
EfficientNetV2S	0.9114	0.9114	0.9114	0.9821
EfficientNetV2L	0.8896	0.8895	0.8895	0.9678
LeafDoc-Net_PReLU	0.9494	0.9494	0.9494	0.9950
LeafDoc-Net_Relu	0.9367	0.9367	0.9367	0.9911
**LeafDoc-Net**	**0.9873**	**0.9873**	**0.9873**	**0.9996**

**Figure 9:**
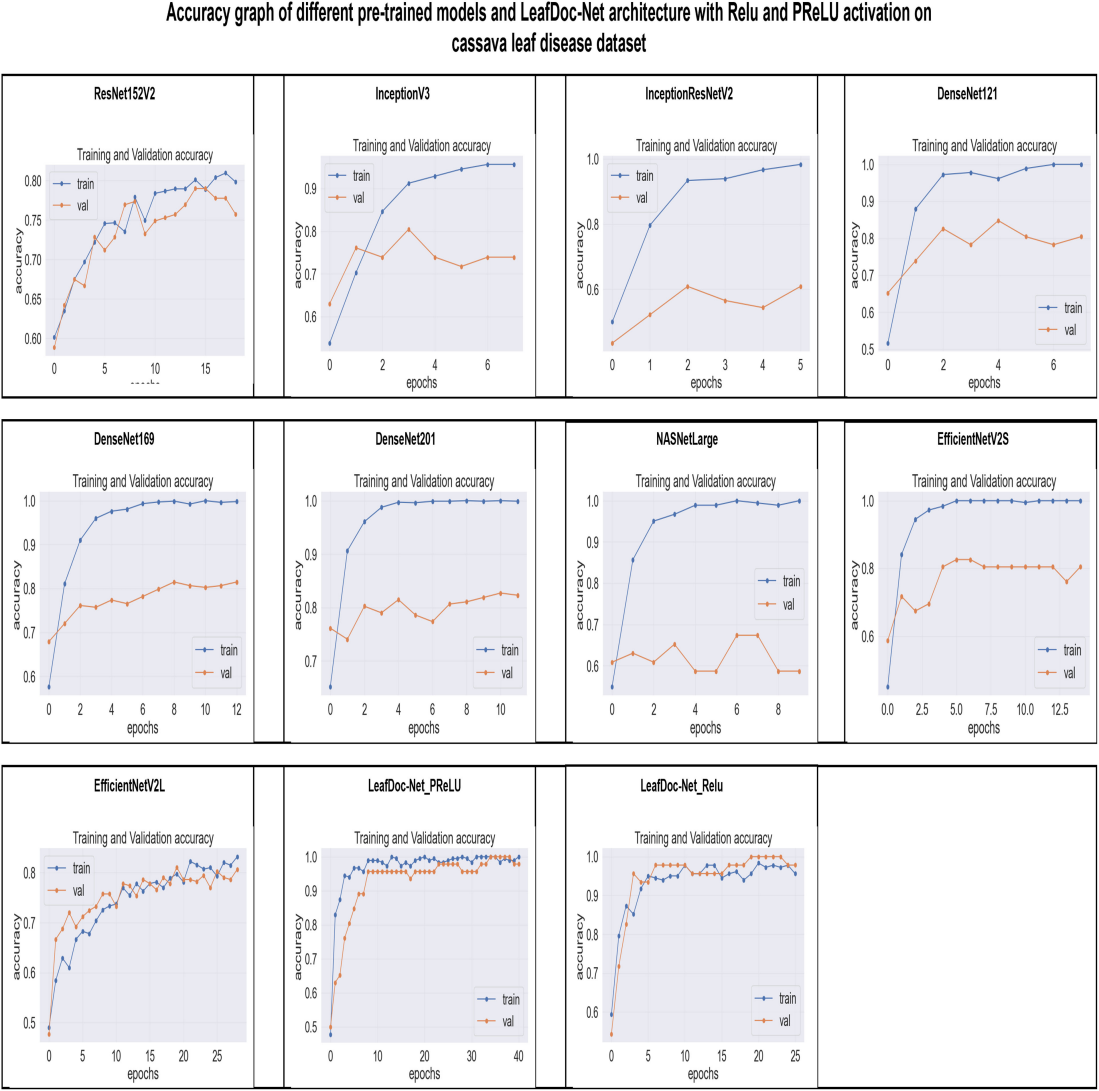
The accuracy graphs of different pre-trained models and the proposed architecture LeafDoc-Net with PReLU and Relu activation on cassava leaf disease dataset.

**Figure 10:**
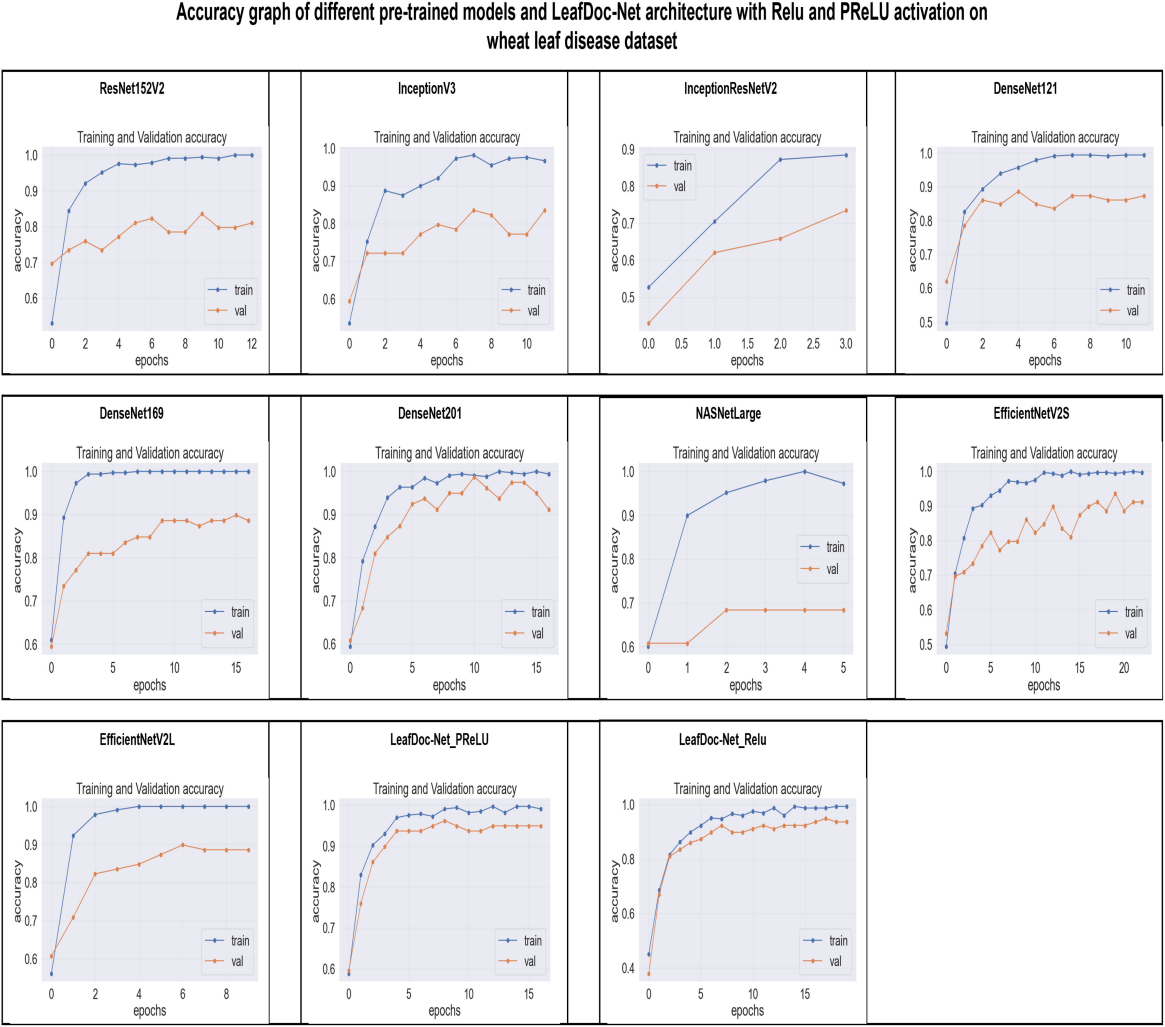
The accuracy graphs of different pre-trained models and the proposed architecture LeafDoc-Net with PReLU and Relu activation on wheat leaf disease dataset.

From the comparison, it is clear that our proposed architecture LeafDoc-Net, showed excellent performance on both datasets, proving its robustness. It is also observed that the activation function dramatically impacts the performance of deep learning models. We experimented with three different activation functions applied to our proposed architecture. The swish activation function outperformed PReLU and Relu in our case. This activation function exhibits 3% better results on average on both datasets than PReLU and Relu. **Figures 9**, **10** represent the accuracy graphs of different pre-trained models and the proposed architecture LeafDoc-Net with PReLU and Relu activation on cassava and wheat leaf disease datasets. The early stopping function was implemented with all the models that stopped the training of the models before 50 epochs if the loss of the models increased for three consecutive epochs to prevent the overfitting problem.

**Figure 11:**
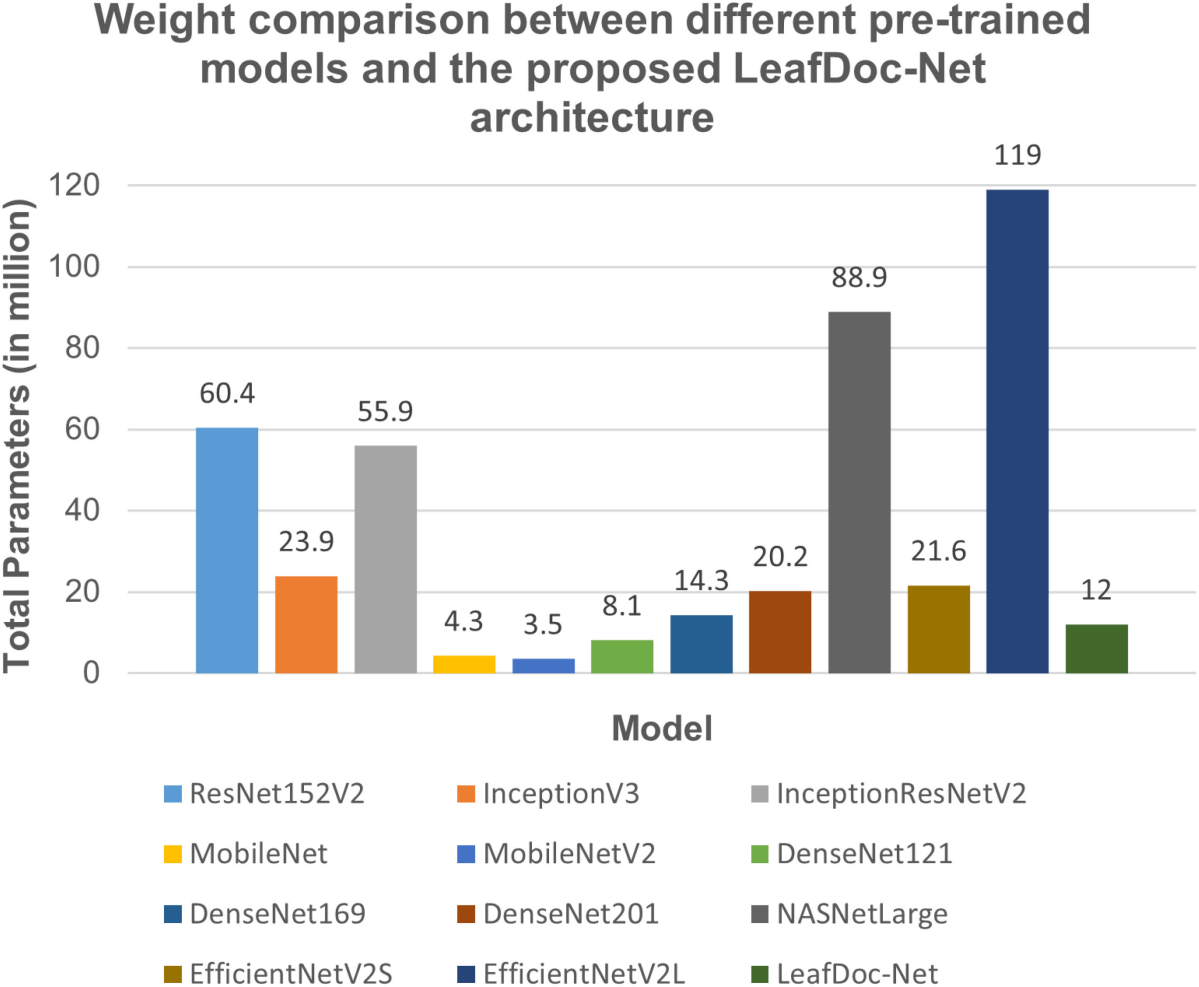
Weight comparison between different pre-trained models and our proposed LeafDoc-Net architecture.

LeafDoc-Net is a lightweight architecture compared to the many pre-trained models we experimented with for this research. We modified two very lightweight pre-trained models, namely DenseNet121, which has only 8.1 million parameters, and MobileNetV2, which has only 3.5 million parameters. Including extra layers with these two models and the concatenation of feature maps from the two models slightly increases our proposed architecture’s weight. It has 12 million parameters, which helped the architecture perform better than any other pre-trained models. The lightweight model takes less training time and can be implemented in low-resource devices, which agriculture requires. **Table 4**, **Figure 11** presents the weight comparison between different pre-trained models and the proposed LeafDoc-Net architecture.

**Table 4:** The comparison of the total parameters of the proposed LeafDoc-Net architecture and experimented pre-trained models.

Model	Total Parameters (in million)
ResNet152V2	60.4
InceptionV3	23.9
InceptionResNetV2	55.9
MobileNet	4.3
MobileNetV2	3.5
DenseNet121	8.1
DenseNet169	14.3
DenseNet201	20.2
NASNetLarge	88.9
EfficientNetV2S	21.6
EfficientNetV2L	119
LeafDoc-Net	12

**Figure 12:**
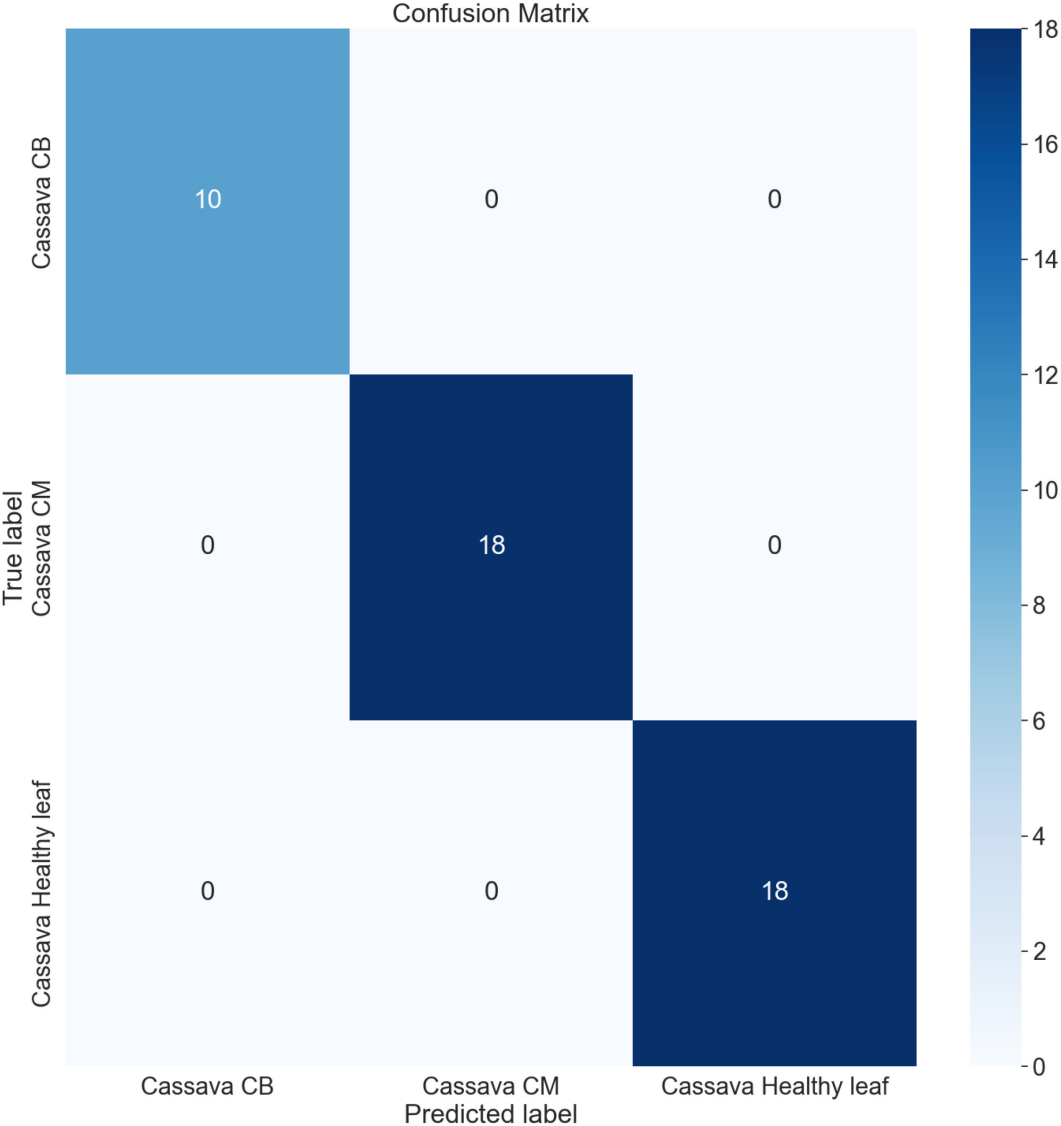
The LeafDoc-Net architecture’s confusion matrix on the cassava leaf disease dataset.

**Figure 13:**
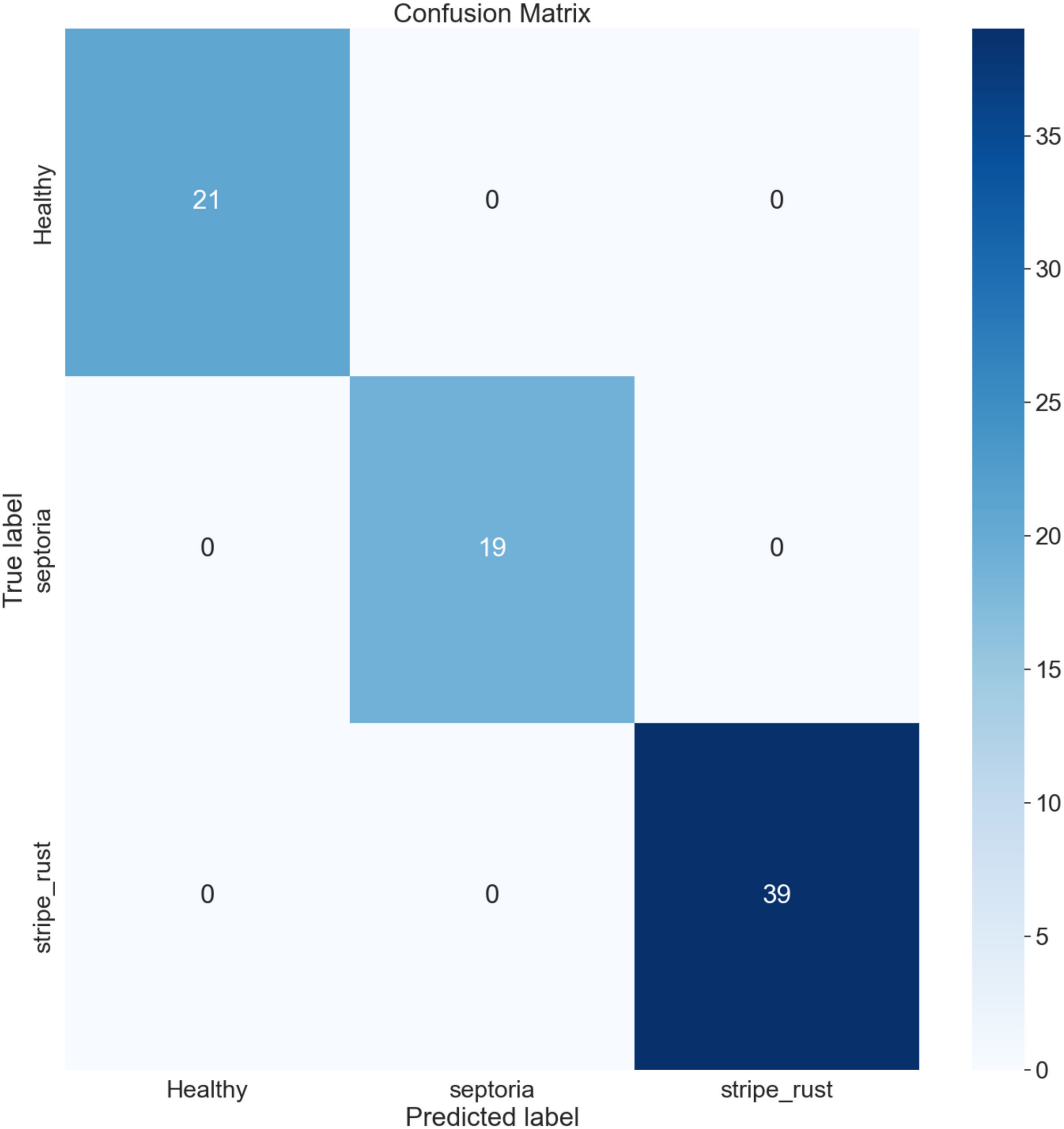
The LeafDoc-Net architecture’s confusion matrix on the wheat leaf disease dataset.

The confusion matrix (CM) has been employed to present an in-depth overview of the performance of the model, as it encompasses various metrics, including accuracy (AC), precision (PR), recall (RE), and AUC. It is organized in a manner where each row and column represent the predictions made for a specific label category compared to the actual labels. It is a comprehensive instrument for accurately analyzing and interpreting the model’s predictions. **Figures 12**, **13** represent the confusion matrix of LeafDoc-Net on cassava and wheat leaf diseases, respectively.

The confusion matrix visually represents the correct predictions indicated by diagonal blue-colored boxes. Among the 46 cassava leaf disease test images dataset, the LeafDoc-Net architecture demonstrated high accuracy by accurately predicting all 46 images. On the other hand, the wheat leaf disease dataset consisted of 79 test images. LeafDoc-Net showed a notable level of accuracy by correctly predicting all 79 images without any instances of misclassification.

**Figure 14:**
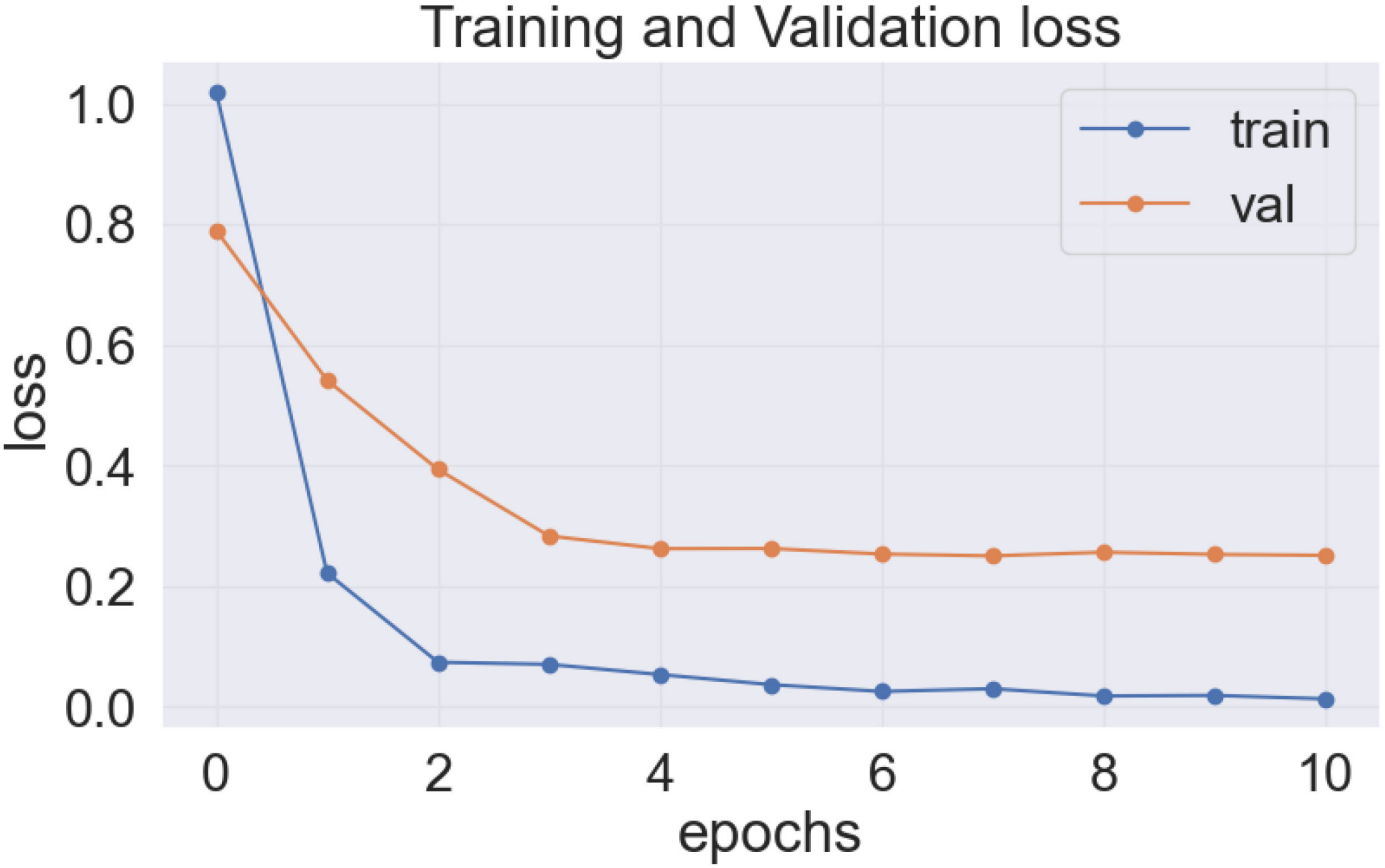
DenseNet201 model’s loss on cassava leaf disease dataset.

**Figure 15:**
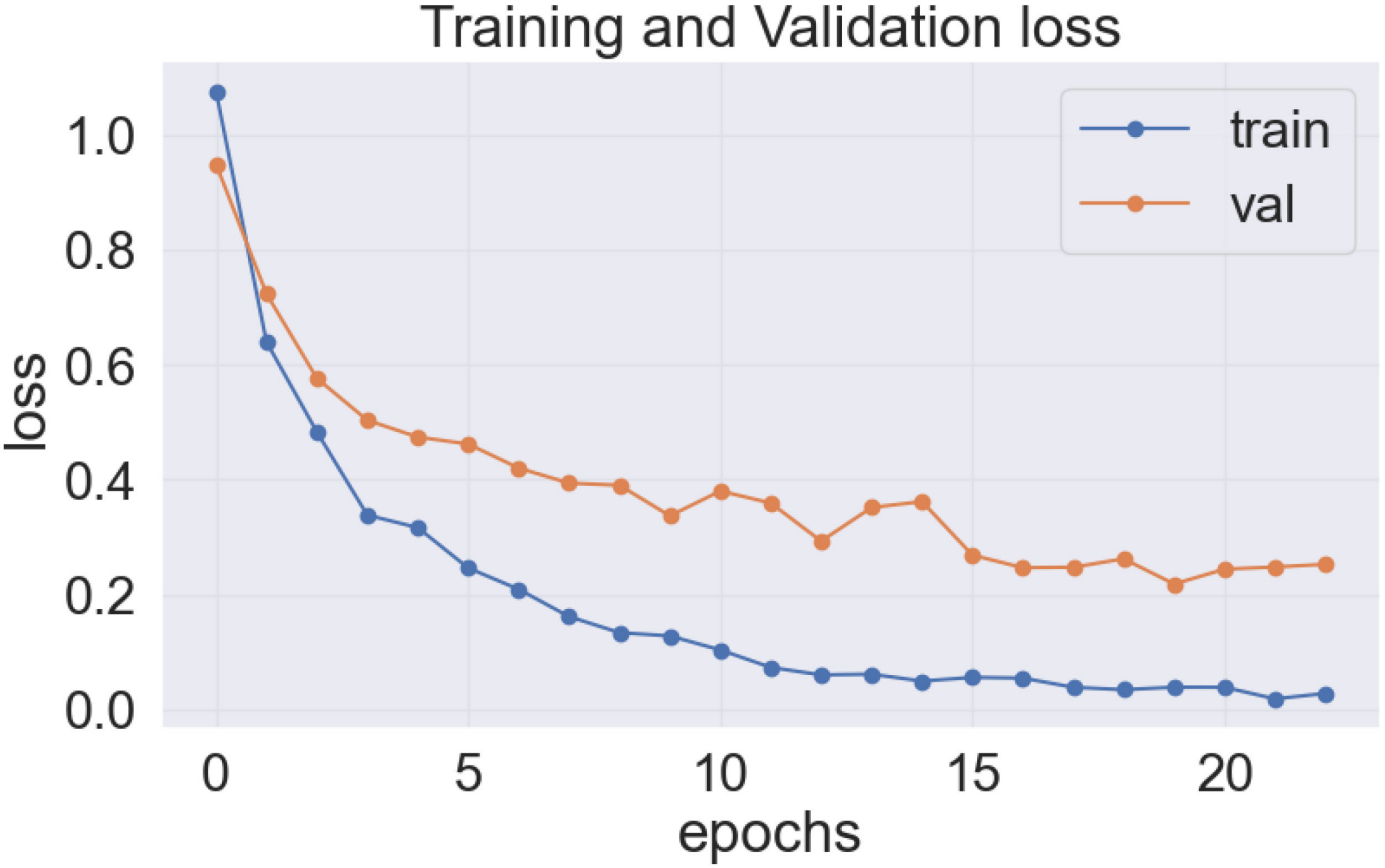
EfficientNetV2S model’s loss on wheat leaf disease dataset.

As previously stated, the best-performing base model utilized for the data sets of the cassava (DenseNet201), and wheat (EfficientNetV2S) leaf disease encountered the issue of underfitting, presented in **Figures 14**, **15**.

In the **Figures 14** and **15**, it was observed that the pre-trained model’s training loss line was much lower than the validation loss indicating the underfitting issue. Early stopping functionality avoids the overfitting issue in the pre-trained model as it stopped the training process earlier than 50 epochs when the validation loss increased.

**Figure 16:**
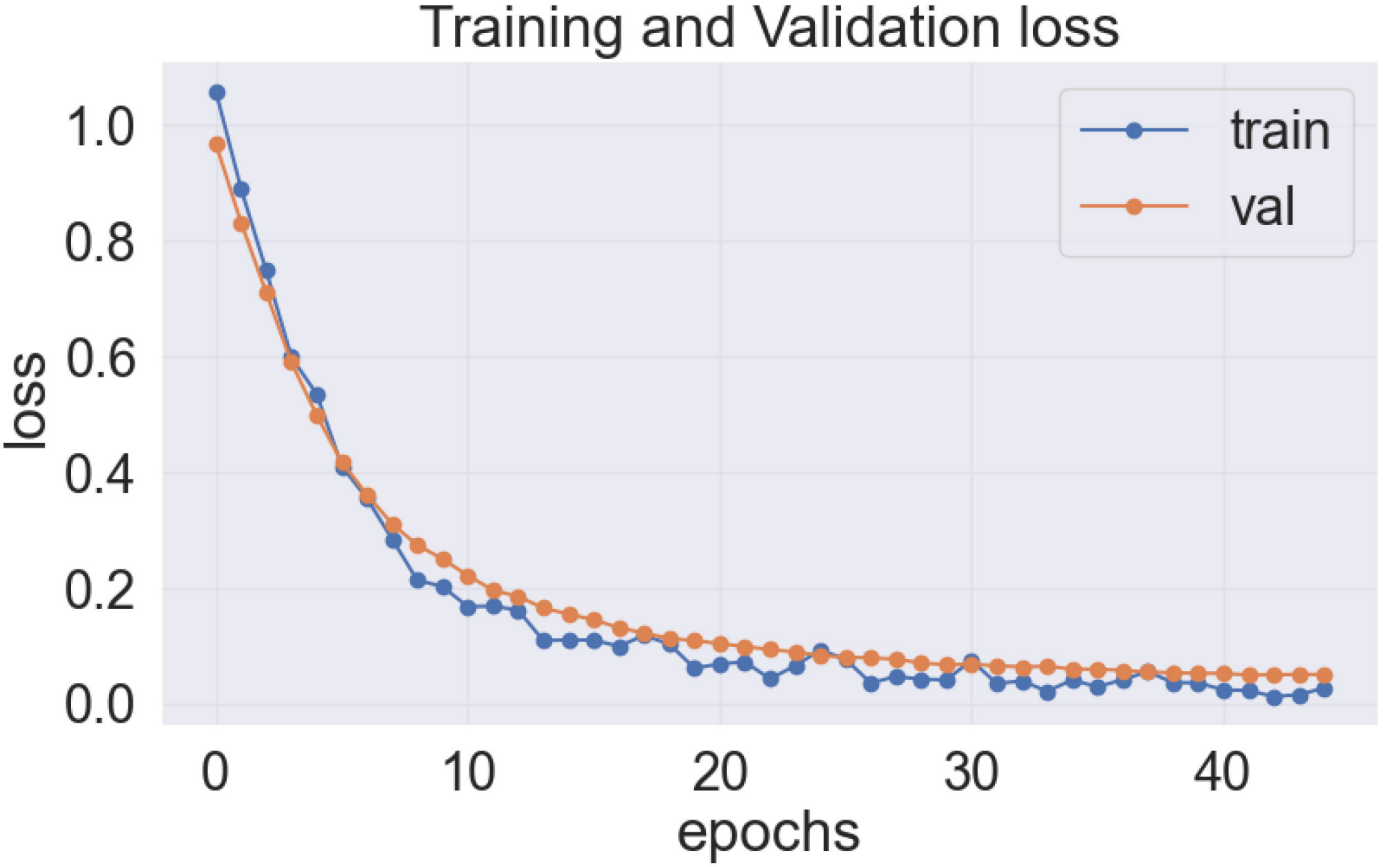
LeafDoc-Net architecture’s loss on cassava leaf disease dataset.

**Figure 17:**
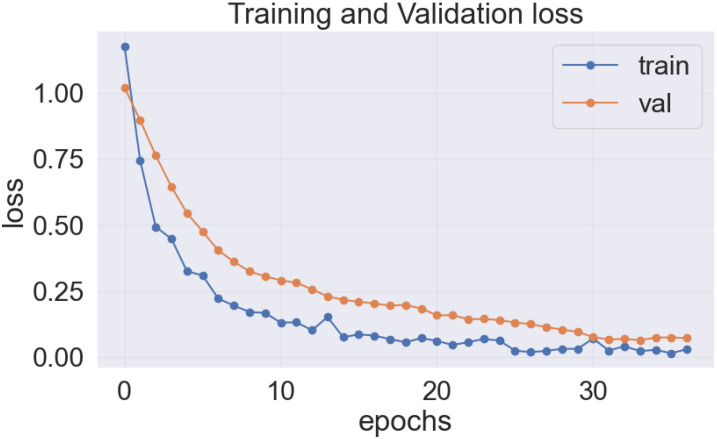
LeafDoc-Net architecture’s loss on Wheat leaf disease dataset.

The proposed LeafDoc-Net addresses the issues of underfitting and overfitting by employing a more complex architecture that involves modifications to DenseNet121 and MobileNetV2. These modifications include incorporating attentive transition, batch normalization, global average pooling, and dense layers with swish activation. Including an early stopping function in the proposed model, they have effectively addressed the overfitting issue by terminating the training process when there was an increase in validation loss. **Figures 16**, **17** depict the graphical representations of the loss for the LeafDoc-Net architecture across the two datasets associated with leaf diseases.

**Figure 18:**
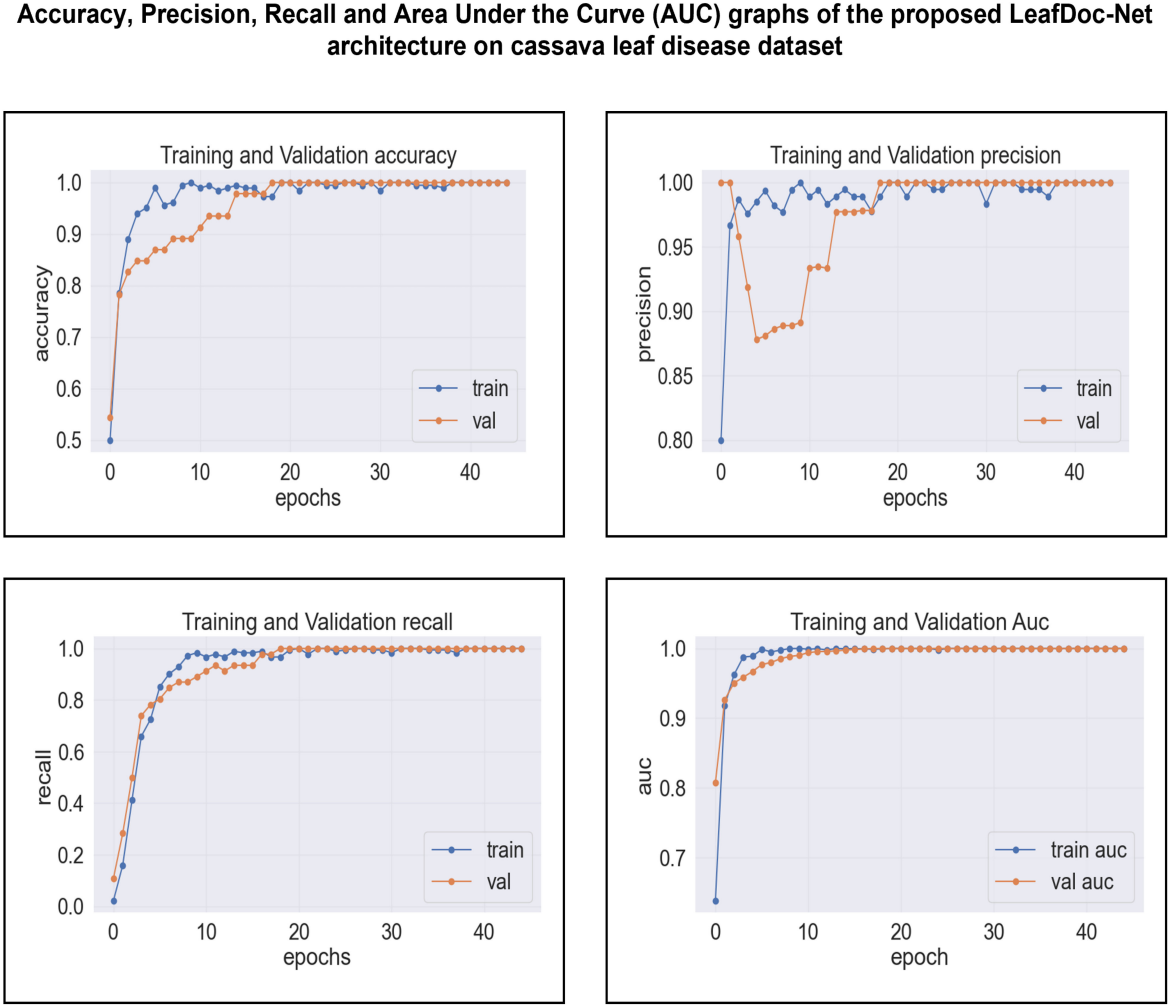
The accuracy, precision, recall and area under the Curve (AUC) graphs of the proposed architecture LeafDoc-Net on cassava leaf disease dataset.

**Figure 19:**
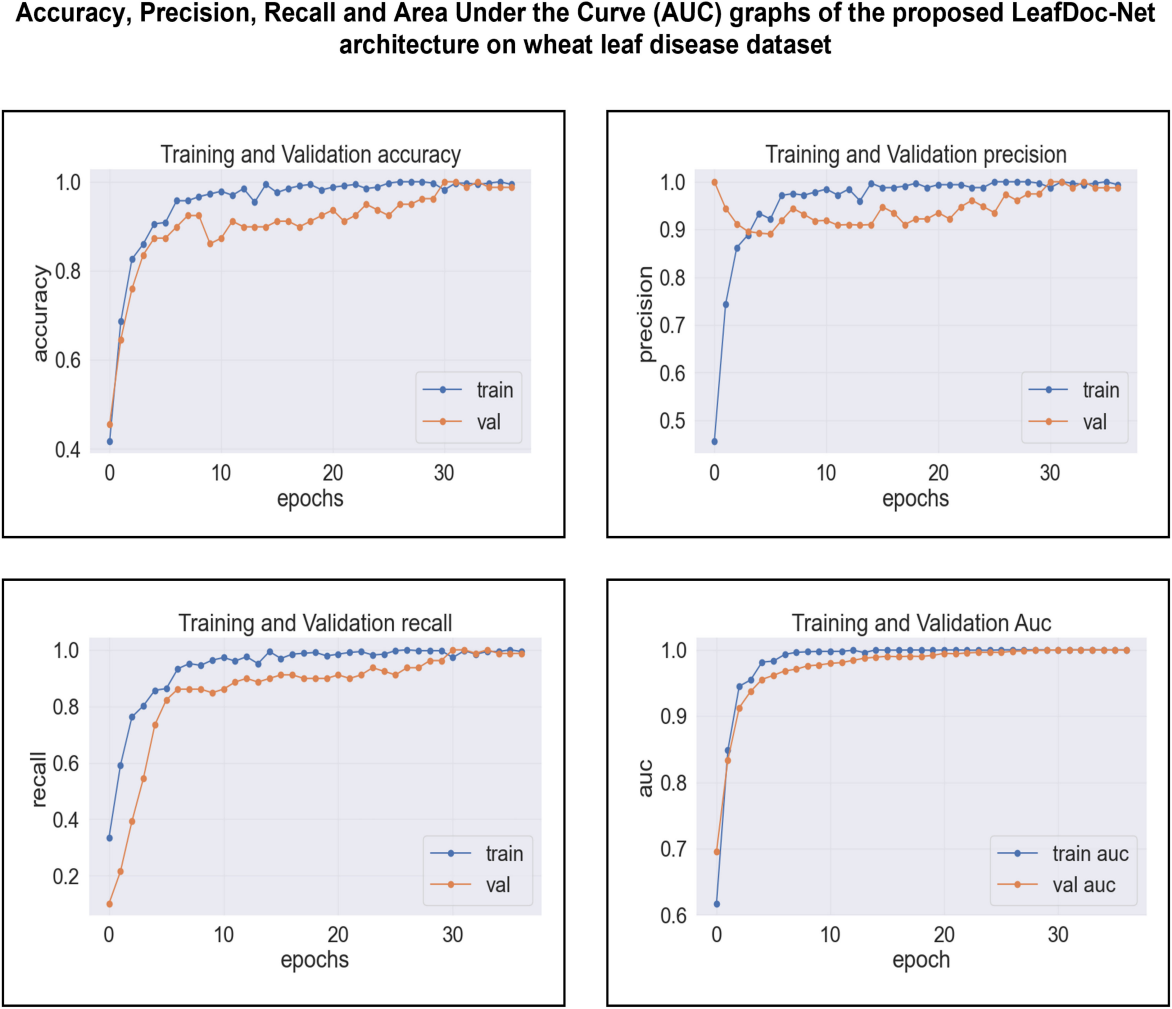
The accuracy, precision, recall, and area under the Curve (AUC) graphs of the proposed architecture LeafDoc-Net on wheat leaf disease dataset.

Based on an analysis of the provided graphs, it can be concluded that the LeafDoc-Net architecture exhibited excellent performance. Initially, the architecture showed a slight underfitting problem, as depicted in **Figures 16**, **17**. Nevertheless, as the curves for training and validation approached a state of convergence, the architecture exhibited enhanced learning capabilities and efficiently displayed its capacity to gain knowledge from the training data. The early stopping function stopped the training process of the proposed architecture when the validation loss started to increase, which prevented the overfitting problem. The loss graphs show a consistently minimal difference between the training and validation loss values, suggesting that the architecture performed effectively and did not encounter any issues related to underfitting and overfitting. **Figures 18**, **19** represent the accuracy, precision, recall, and area under the Curve (AUC) graphs of the proposed architecture LeafDoc-Net on both cassava and wheat leaf disease datasets.

**Figure 20:**
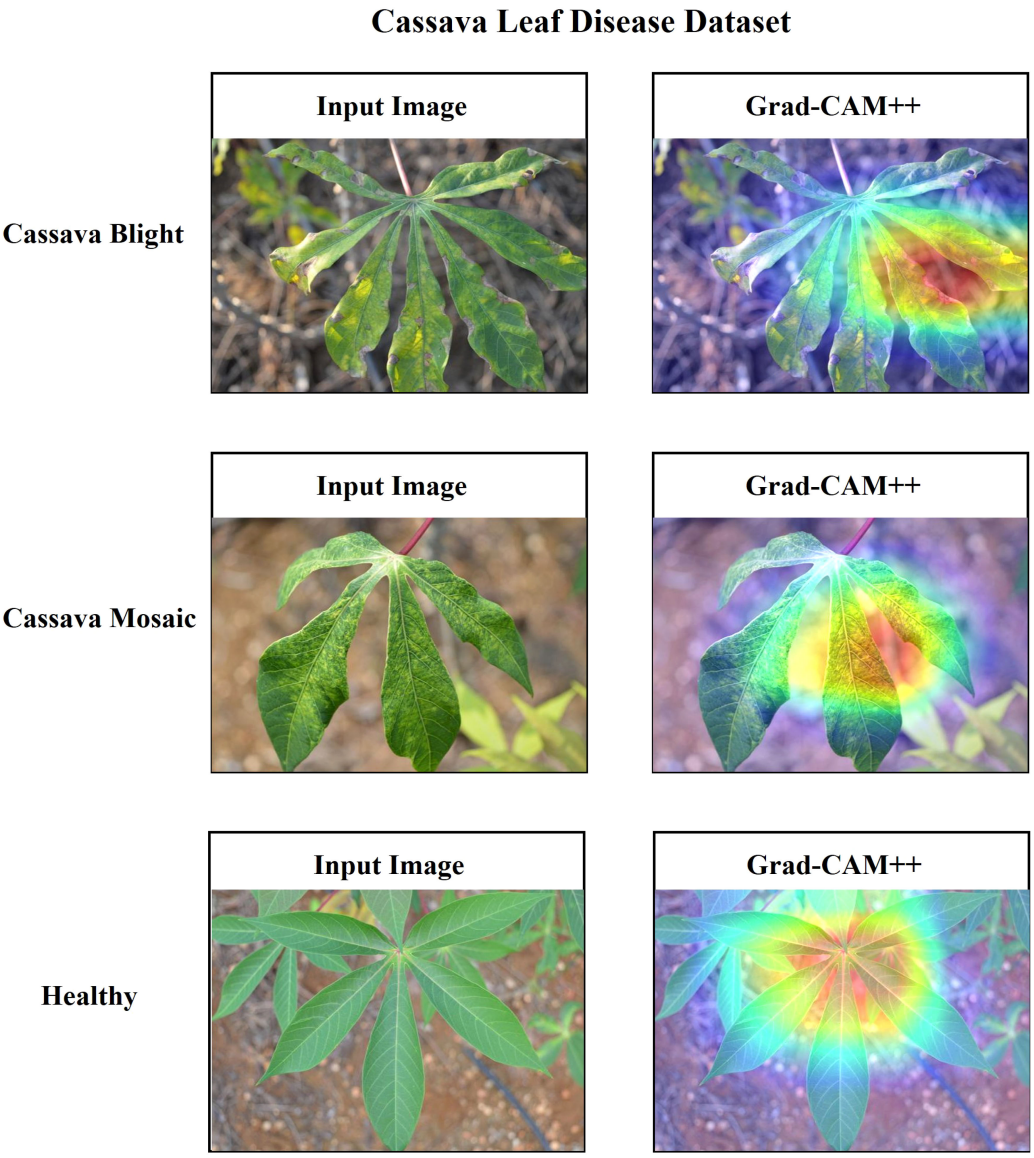
Input Image vs. Grad-CAM++ assisted output of different classes of cassava leaf disease dataset.

**Figure 21:**
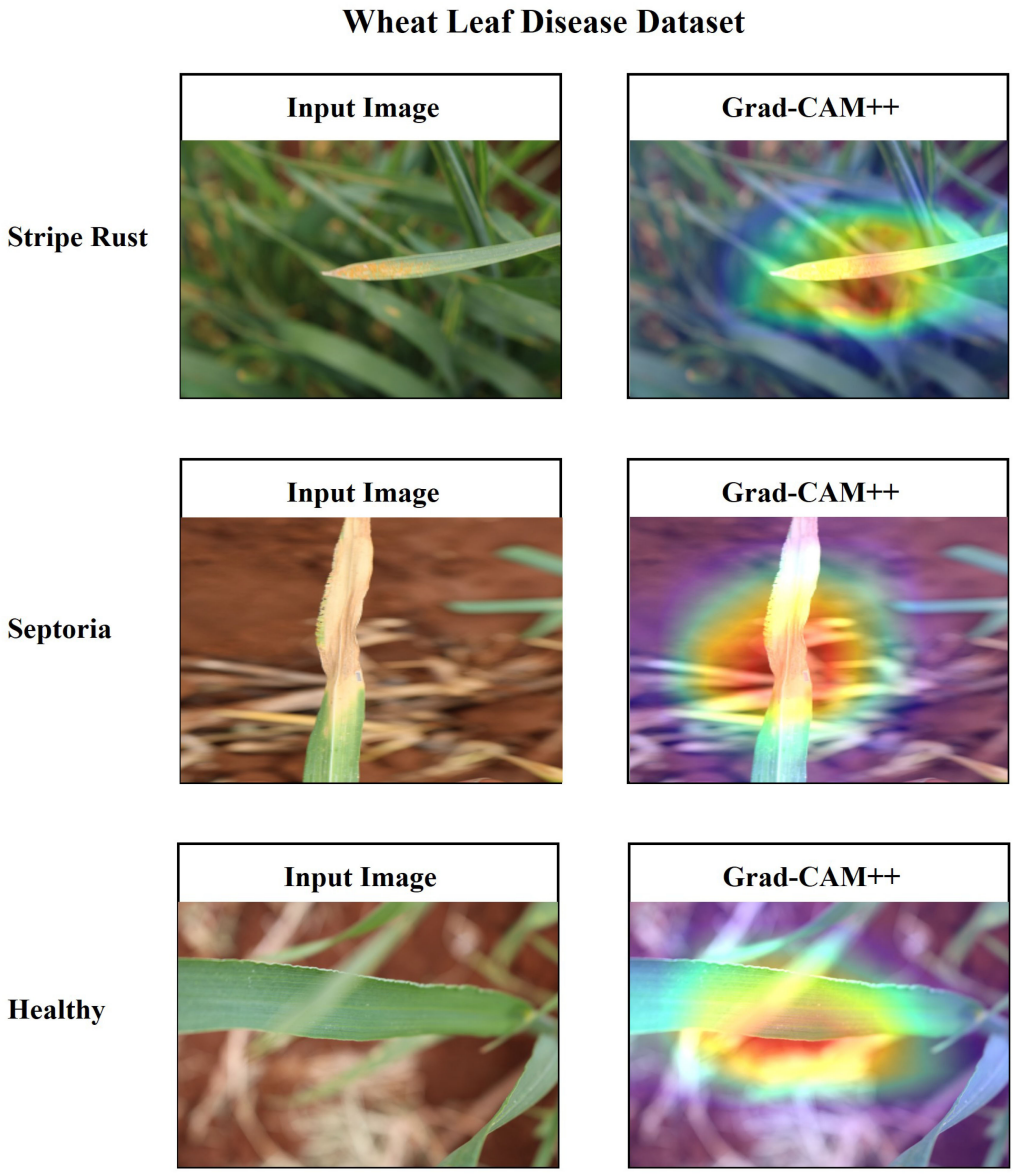
Input Image vs. Grad-CAM++ assisted output of different classes of wheat leaf disease dataset.

Additionally, it is essential to establish a sense of trust among users by providing a comprehensive explanation of the suggested architecture. In this research, the utilization of Grad-CAM++ has been employed to visually represent the proposed architecture’s predictions based on the specific regions of the image on the ‘conv_2d’ layer of the architecture. Grad-CAM++ was utilized to capture and analyze every image from each class within the dataset. Each image presents a unique set of challenges, including variations in rotation and background. The generated region exhibits a deep red hue, indicating its significance as the primary location determined by the learned model and predicted label. The heatmap has revealed the presence of a red spot in each area where a defect is present. The contours and edges have been accurately visualized, exhibiting a lack of overlapping concerns. **Figures 20**, **21** present the input and Grad-CAM++ generated images from all four-leaf disease datasets.

## 5 Discussion

This study investigated multiple pre-existing models based on the transfer learning concept for accurate leaf disease detection. All the experimented pre-trained models struggled to deliver the expected results. Nevertheless, it was noted that every model, including DenseNet121 and MobileNetv2, experienced underfitting challenges when dealing with a restricted number of complex images from the datasets. In general, transfer learning-based models exhibit enhanced performance when trained on extensive datasets, as they can acquire more features from the data. Some heavyweight pre-trained models required longer to be trained on the selected datasets due to the vast number of learning parameters. To address these particular challenges, we proposed a solution known as LeafDoc-Net within the context of this study. The LeafDoc-Net architecture successfully addressed the issues of fewer complex images and underfitting across various datasets and demonstrated exceptional performance. Integrating complex layers helped the proposed architecture learn more features from the less complex images. As a result of its complex architectural design, LeafDoc-Net possessed the capacity to acquire noise during the training process. We utilized efficient data preprocessing and augmentation methods to address the potential overfitting issue. LeafDoc-Net is a lightweight architecture that takes very little time to be trained and can be implemented in any low-resource computational device. In general, the LeafDoc-Net framework introduced an innovative strategy to address the issue of underfitting in the context of datasets containing a limited number of complex images.

## 6 Conclusion and the future work

Plants have an unparalleled connection with every living creature, primarily due to their crucial role in survival and finances. The health of plants plays a crucial role in agricultural productivity and can result in substantial economic losses. The presence of infectious, bacterial, fungal, and insect-related pathogens can significantly negatively impact the condition and health of plant leaves. Early diagnosis of these diseases is crucial for farmers to implement timely treatments. This research aimed to establish a lightweight and robust method for detecting plant leaf diseases, regardless of leaf size or background complexity, to aid farmers in detecting such diseases. This study examined several advanced transfer learning techniques. Nevertheless, most pre-trained models exhibited poor performance with underfitting problems during the training process because of the less amount of input data. To effectively tackle these issues, and achieve optimal performance within a single network, a lightweight and robust transfer learning-based architecture called LeafDoc-Net was proposed. The performance of this architecture was enhanced by incorporating various techniques. LeafDoc-Net effectively addressed these challenges and demonstrated superior performance to existing models regarding the accuracy, precision, recall, and area under the curve (AUC) performance metrics across the two datasets encompassing cassava, and wheat leaf diseases. Although the LeafDoc-Net architecture demonstrated encouraging outcomes in generic leaf disease detection, further investigation is necessary to enhance and expand the abilities of leaf disease detection models within a more comprehensive framework. Our possible research attempts will go deeply into further investigation of optimization techniques like increasing model complexity, variation in regularization, learning rate, optimizer, and loss function to mitigate the problems of overfitting and underfitting and will be implemented into the proposed architecture. By balancing model complexity and generalization, these strategies will increase the architecture’s capacity to generalize successfully to new, unknown data by reducing the likelihood of underfitting and overfitting. Our objective will be to present an enhanced and accurate architecture for detecting multi-leaf diseases. By recognizing the constraints of existing research and utilizing advancements in deep learning techniques, we aim to contribute to the effective management of leaf diseases in various plant species.

## Data availability statement

Publicly available datasets were analyzed in this study. This data can be found here: https://data.mendeley.com/datasets/3832tx2cb2/1; https://data.mendeley.com/datasets/wgd66f8n6h/1.

## Author contributions

MM: Conceptualization, Methodology, Data Curation, Formal analysis, Software, Resource, Visualization, Writing – original draft, Writing – review & editing. MFM: Supervision, Validation, Visualization, Writing – review & editing. SA: Funding acquisition, Writing – review & editing. MS: Formal analysis, Writing – original draft. M-AJ: Investigation, Writing – original draft. DC: Supervision, Writing – review & editing.
