# Supplementary figures and images for: A robust and light-weight transfer learning-based architecture for accurate detection of leaf diseases across multiple plants using less amount of images
Source: Front Plant Sci. 2024 Jan 11;14:1321877. doi: 10.3389/fpls.2023.1321877 (PMC10809160; doi:10.3389/fpls.2023.1321877)

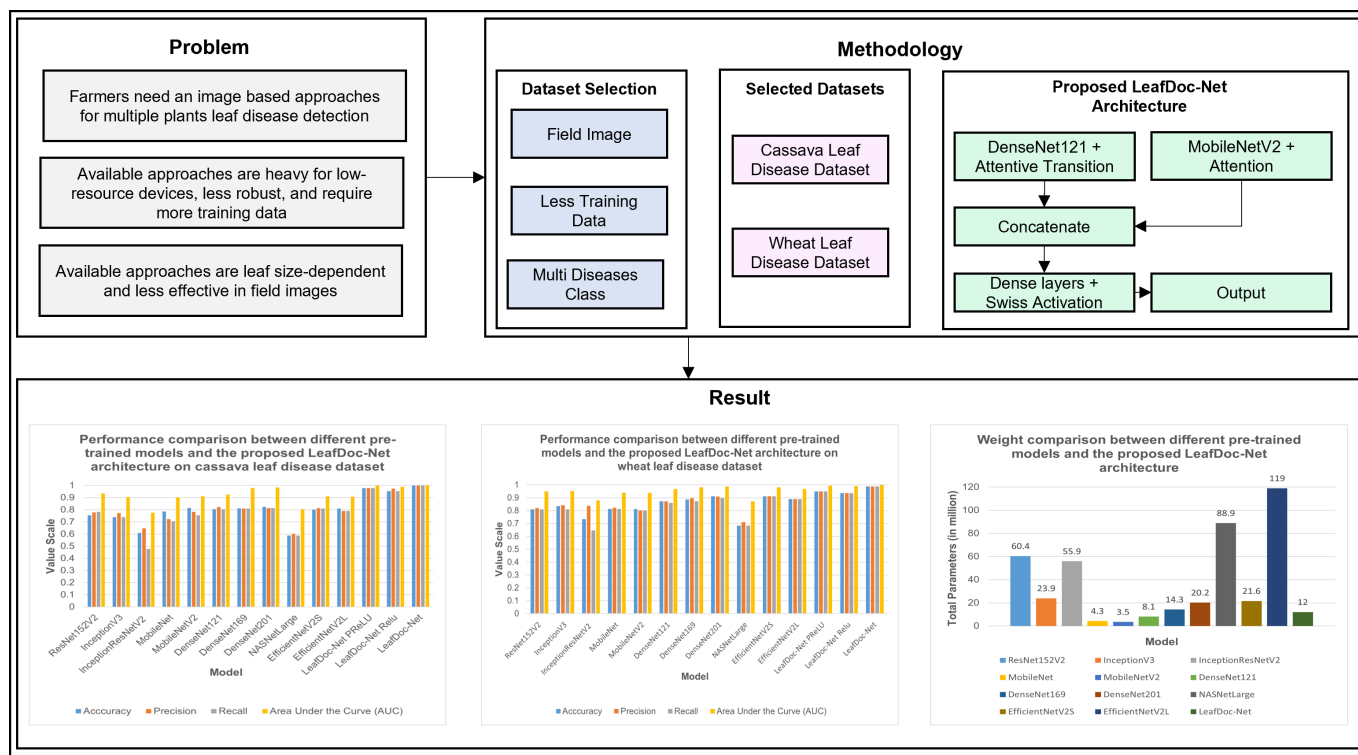

**Figure S1.** A visual representation of the problems, the methodology, and the results.

Supplement: Supplementary file 1 [file DataSheet_1.pdf]
